# Interfacial Engineering by Metallic Ions and Organic Ammonium Ligand Passivation for Perovskite Solar Cells

**DOI:** 10.1002/advs.202520257

**Published:** 2026-04-09

**Authors:** Abraham Adenle, Purevlkham Myagmarsereejid, Selengesuren Suragtkhuu, Tuul Tsagaantsooj, Solongo Purevdorj, Eric Campbell, Sina Jamali, Oisín E. FitzGerald, Taylor J. Z. Stock, Thomas J. Macdonald, Joseph F. S. Fernando, Hui Jin, Paul E. Shaw, Michitoshi Hayashi, Batjargal Sainbileg, Chihaya Adachi, Yu Lin Zhong, Munkhbayar Batmunkh

**Affiliations:** ^1^ School of Environment and Science Griffith University Nathan Queensland Australia; ^2^ Center for Organic Photonics and Electronics Research (OPERA) Kyushu University Fukuoka Japan; ^3^ Department of Electronic and Electrical Engineering University College London London UK; ^4^ London Centre For Nanotechnology University College London London UK; ^5^ Centre for Microscopy and Microanalysis The University of Queensland St Lucia Queensland Australia; ^6^ School of Chemistry and Physics and Centre for Materials Science Queensland University of Technology (QUT) Brisbane Queensland Australia; ^7^ Centre for Organic Photonics & Electronics, School of Chemistry and Molecular Biosciences The University of Queensland Brisbane Queensland Australia; ^8^ Center for Condensed Matter Sciences, Center of Atomic Initiative For New Materials National Taiwan University Taipei Taiwan

**Keywords:** interfacial engineering, perovskite solar cells, photovoltaic, surface passivation

## Abstract

Surface passivation is essential for minimizing ion vacancies and trap centers in perovskite crystals, but the mechanisms governing enhanced interfacial charge separation and suppressed recombination remain an active area of research. In this study, a synergistic post‐treatment approach employing phenethylammonium iodide (PEAI) and antimony iodide (SbI_3_) is introduced, which not only passivates the traps but also facilitates the charge transportation at the interface. By using advanced characterization techniques, we confirm the dual incorporation of PEAI and Sb^0^, revealing improved charge carrier dynamics and surface passivation. The *p*‐type metallic Sb doping, suitable band energy alignment, reduced defect density, and minimized surface ion vacancies collectively enhance the interfacial charge separation, thus resulting in an enhanced power conversion efficiency (PCE). Notably, unencapsulated devices with PEAI+Sb treatment demonstrated impressive thermal stability, retaining over 80% of the initial PCE after 5 h of continuous heating at 85°C temperature under N_2_ condition. This theoretical findings confirm that Sb preferentially binds to the PEA phenyl ring, yielding a stable interfacial configuration, stabilizing the surface and enhancing the interfacial charge transport. This work presents an effective strategy and provides insights into charge modulation using metallic ions, contributing to improved device efficiency and stability in perovskite solar cells.

## Graphical abstract

A synergistic approach using phenethylammonium iodide (PEAI) and antimony iodide (SbI_3_) enhances the operational stabilities of perovskite solar cells by providing p‐type doping effect, suitable band energy alignment, reduced defect density, and minimized surface ion vacancies in the perovskite films.

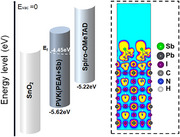

## 1 Introduction

Perovskite solar cells (PSCs) have emerged as a frontrunner in next‐generation photovoltaic (PV) technologies due to their high power conversion efficiencies (PCEs) and low fabrication cost [1, 2, 3]. Charge carrier dynamics, recombination processes and interface engineering play key roles in achieving high PCEs and robust devices [4, 5]. Enhancing charge extraction and minimizing interfacial defects are crucial for improving both the efficiency and stability of PSCs [6, 7, 8]. Therefore, achieving precise control over interfacial charge carrier injection to reduce non‐radiative recombination and promote charge separation at the buried interfaces of perovskite (PVK) films is of great importance. Over the past decades, strategies such as surface passivation [9, 10, 11, 12], additive engineering [13, 14, 15, 16], interfacial defect passivation [17, 18, 19, 20], and reducing surface halide deficiency via metal halides modification [21] have been developed to modify the physicochemical and optical properties of metal halide perovskites. These strategies aim to facilitate the separation of photogenerated electrons and holes, while reducing the surface recombination, thus enhancing solar‐to‐electrical conversion efficiency.

Among these techniques, surface passivation using organic ammonium halide ligands has been proven to be the dominant approach for defect passivation and ion vacancy treatment. A wide range of organic halide salts such as halogenated phenethylammoniums, propylamine hydroiodide (PAI), phenethylammonium iodide (PEAI) and 1,3‐diaminopropane dihydroiodide (PDADI) have shown great promises in passivating perovskite surface defects [22, 23]. In particular, PEAI has garnered much attention for its unique ability to form quasi‐2D perovskite layers, which are vital for enhancing PSC performance by effectively passivating surface and grain boundary defects [24, 25, 26, 27, 28]. However, the long‐term stability of PEAI‐passivated interfaces poses a significant challenge [29, 30, 31]. For example, Jiang et al. reported improvements in solar cell efficiency by reducing surface interface defects and inhibiting non‐radiative recombination through PEAI application [24]. Despite their success in reducing surface defects and enhancing PV efficiencies, a major hurdle was the chemical deprotonation of the ammonium group in PEAI. This process, caused by environmental factors such as moisture and thermal stress, compromises the structural integrity of the passivation layer, weakens defect passivation and deteriorates interfacial charge extraction capabilities, leading to poor device stability [32, 33]. Therefore, addressing this key challenge is crucial for maintaining the beneficial effects of surface passivators for high efficiency and stable PSCs. By stabilizing the PEAI layer and preserving its protonated state, it is possible to sustain effective defect passivation and optimal charge dynamics over extended period of operation, thereby promoting the long‐term stability of PSCs. More importantly, a comprehensive understanding of interface charge modulation and the chemical stability of passivation ligands such as PEAI is essential for unlocking robust and high‐performance PV devices.

In this work, we present an in‐situ post‐treatment and interfacial charge modulation strategy employing PEAI and antimony (III) iodide (SbI_3_) to simultaneously passivate α‐FAPbI_3_ perovskite and introduce metallic antimony (Sb^0^) as an efficient interfacial charge modulation, suppressing interface non‐radiative recombination and enhancing device stability. A combination of advanced characterization techniques and theoretical calculations was used to investigate the modified α‐FAPbI_3_ films and devices. Furthermore, we realized that the interfacial charge separation was facilitated by the p‐type doping effect, suitable band energy alignment, reduced defects and minimized surface ion vacancies. Remarkably, the unencapsulated devices with PEAI+Sb treatment demonstrated significantly enhanced stability, retaining over 80% of its initial performance after 5 h of continuous heating at 85°C under N_2_ condition.

## 2 Results and Discussion

### 2.1 Fabrication of Perovskite Films and Interfacial Modification

**FIGURE 1:**
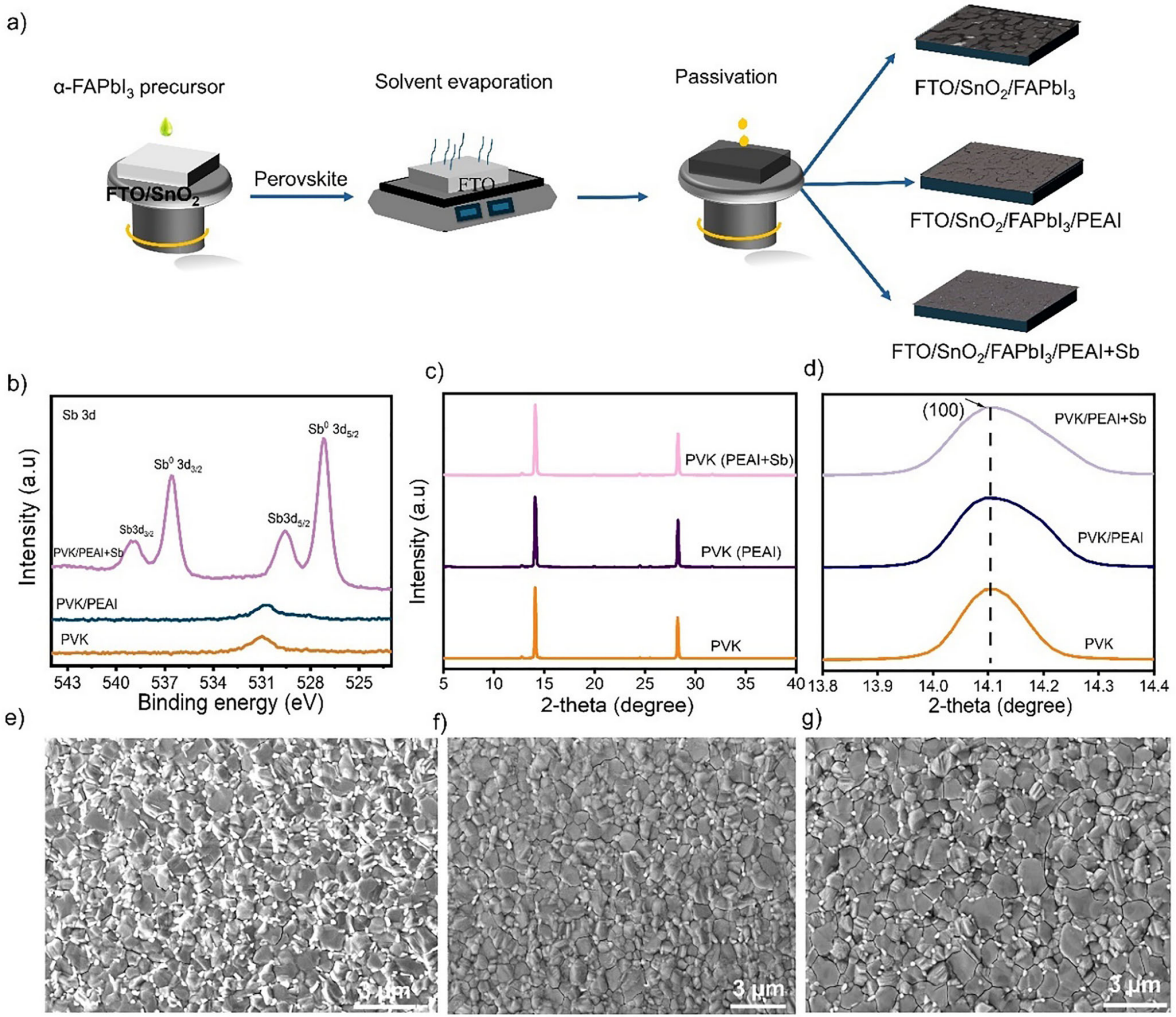
Interfacial modulation and surface characterization. (a) A schematic illustration of the perovskite fabrication process using PEAI and PEAI+Sb surface passivation, (b) high‐resolution (HR) XPS Sb 3d spectra of the perovskite films, (c) wide and (d) magnified (100) XRD patterns of the FAPbI_3_ perovskite (PVK) films. (e–g) Top‐view SEM images of the pristine PVK, PVK(PEAI), and PVK(PEAI+Sb) films.

Figure 1a depicts the fabrication process of perovskite films deposited on tin (IV) oxide coated fluorine‐doped tin oxide (FTO) substrates, followed by interfacial passivation. Comprehensive details of the experimental procedures, including materials preparation and film deposition parameters, are provided in the Supporting Information. In brief, the perovskite precursor solution was formulated at a concentration of 1.4 M by mixing α‐formamidinium lead iodide (α‐FAPbI_3_) powder with methylammonium dichloride (MDACl_2_) at 3.8 mol% and methylammonium chloride (MACl) at 35 mol%, all dissolved in a mixed solvent of dimethylformamide (DMF) and dimethyl sulfoxide (DMSO). The resulting precursor mixture was subsequently filtered into glass vials to ensure homogeneity and remove any particulate impurities prior to film deposition. Meanwhile, the passivation solutions were prepared by dissolving PEAI and SbI_3_ in isopropanol. The as‐prepared PEAI and PEAI+Sb solutions were deposited onto the perovskite films. Three main films including FTO/SnO_2_/PVK, FTO/SnO_2_/PVK/(PEAI), and FTO/SnO_2_/PVK/(PEAI+Sb) were fabricated and used for device fabrication. However, prior to deposition, the ratio of SbI_3_ incorporated into the PEAI+Sb passivation solution was optimized to achieve the best performance and interface quality, as specified in Figure S1. It is noteworthy that this optimization process is critical for achieving the appropriate balance between effective defect passivation and preservation of the perovskite structural integrity since high Sb concentration could lead to either insufficient passivation or undesired alteration in surface chemistry.

To confirm the presence of metallic antimony (Sb) on the surface of PVK films, we employed X‐ray photoelectron spectroscopy (XPS) to characterize the elemental composition and chemical states of both pristine and passivated perovskite samples. As shown in Figure 1b, the XPS spectra reveal noticeable differences in the Sb 3d_5/2_ region between the PEAI+Sb passivated films and non‐passivated (pristine) perovskite films. The appearance of characteristic peaks corresponding to metallic Sb^0^ confirms the successful formation and presence of metallic antimony on the perovskite surface following the passivation treatment. These metallic Sb^0^ signals are absent in the non‐passivated films, indicating that the Sb species originate specifically from the PEAI+Sb interfacial modification. The observation of Sb predominantly at the perovskite interface is significant and indicates possible functions in regulating the surface energetics and improving interfacial charge transfer processes. Based on our hypothesis, metallic Sb^0^ mediates the charge carrier transportations and facilitates defect passivation. Additionally, precisely discovering Sb oxidation states using XPS provides an in‐depth insight into the chemical states of the perovskite surfaces. By comparing the Sb 3d fitting peaks of the pristine and passivated perovskite films, Sb 3d_5/2_ spectrum of the PEAI+Sb treated perovskite reveals two characteristic peaks at 529.6 eV and 527.2 eV. Notably, the pronounced peak at 527.2 eV provides a strong evidence for the presence of metallic Sb^0^ at the perovskite interface [34]. Additionally, XPS analysis was also performed on perovskite films passivated exclusively with SbI_3_ dissolved in isopropanol (IPA), without PEAI. As illustrated in Figure S2, the SbI_3_ treated film exhibited a dominant Sb 3d_5/2_ peak at 530.2 eV, along with minor peaks at 532.11 and 527.84 eV. These shifts indicate different chemical states of Sb compared to the PEAI+Sb treatment, suggesting that the presence of PEAI influences the chemical environment and oxidation states of Sb at the interface. These signals can be attributed to the presence of Sb^3^^+^ ions in Sb_2_O_3_, Sb^5^^+^ in Sb_2_O_5_, and a trace amount of metallic Sb^0^ [35]. In addition, to confirm the presence of PEAI and metallic Sb^0^ as distinct entities within the PEAI+Sb treated perovskite films, HR C 1s analysis was performed. The characteristic C‐C and C‐N peaks were observed at binding energies of 284.7 and 286.4 eV, respectively, in both PEAI and PEAI+Sb treated films (Figure S3). Moreover, it is important to identify the presence of peak at 292.1 eV, which could be attributed to the π–π* interaction associated with the phenyl group in PEAI cations, to further confirm the successful passivation of PEAI at the perovskite interface. Nevertheless, as shown in Figure S3, the peaks at ∼292.1 eV were not distinguishable from the noise within the signal‐to‐noise ratio. However, the presence C‐C and C‐N peaks located at 284.6 and 286.1 eV, respectively, for PEAI treated samples confirms the presence of PEAI passivation at the perovskite interface [9]. In addition, a prominent C = O peak at 288.1 eV was observed in the control (PVK) film, which is associated with oxygen and moisture. This peak diminished after the PEAI and PEAI+Sb modifications, indicating that the PEAI passivation effectively reduces the degradation of the perovskite layer [9, 36]. These results provide considerable proofs for the simultaneous presence of organic PEAI ligands and metallic Sb^0^ at the perovskite surface. Notably, we used density‐functional theory (DFT) calculations employing α‐FAPbI_3_ structure to investigate the interactions between the passivation agents and the perovskite, as well as the effects of interfacial modification on the physicochemical characteristics of the perovskite films [37]. More importantly, the presence of metallic Sb^0^ is crucial as it has the potential to operate as an interfacial charge mediator, thereby improving the charge transport and extraction at the perovskite interface [38, 39].

Furthermore, X‐ray diffraction (XRD) analysis was performed to examine the interaction between the surface passivator and the perovskite crystal structure. XRD patterns of the perovskite films exhibited the characteristic cubic α‐black phase, with prominent diffraction peaks observed at 14.03° and 28.3°, corresponding to the (001) and (002) crystal planes, respectively (Figure 1c) [40]. Both PEAI and PEAI+Sb treated films exhibited no additional diffraction peaks as compared to the pristine PVK film, indicating that the overall crystal structure remained unchanged. However, a broadening of the (001) diffraction peak was observed in the treated samples, suggesting modifications in crystallinity without any significant shift in their peak positions (Figure 1d). This peak broadening may reflect slight changes in crystal domain size or increased macrostrain within the lattice, likely resulting from the surface passivation process. This indicates that interfacial modification through metallic Sb^0^ and PEAI passivation effectively alters the surface characteristics while preserving the underlying crystal structure and lattice arrangement. Furthermore, the intensity ratio between the (001) and (002) diffraction peaks remain consistent across all samples, highlighting that surface passivation does not significantly impact the orientation of crystalline planes or the structural and optical properties of the perovskite films. In addition, it is noteworthy that from the XRD result, no obvious 2D perovskite peak can be observed even though numerous reports have shown that PEAI can induce the formation of 2D Ruddlesden‐Popper perovskites on the 3D perovskite surfaces. Nevertheless, in our work, PEAI is applied as a very thin layer, low‑concentration post‑treatment and being co‑processed with SbI_3_. Under these conditions, XRD does not reveal the characteristic low‑angle reflections of 2D phases (*n* = 1–2), suggesting that no thick, long‑range ordered 2D perovskite layer is formed. Instead, the PEAI+Sb treatment form a mixed‑dimensional interfacial region, in which PEAI and Sb species locally modify and passivate the surface. This configuration preserves the beneficial defect passivation and band‑alignment effects associated with PEAI‑based interfaces, while avoiding the transport limitations often introduced by thick 2D capping layers. Overall, the XRD analysis confirms that surface passivation with PEAI and PEAI+Sb does not induce notable structural changes within the perovskites, thereby maintaining the integrity of their crystalline framework while improving the surface properties.

Furthermore, to gain deeper insight into the presence of metallic Sb^0^, we conducted time‑resolved depth‐profile XPS. As shown in Figure S4a, the depth‐profile XPS analysis displays the presence of metallic Sb^0^ with a relatively high intensity as compared to Sb^3^^+^ at the initial stage of the measurement. This suggests that metallic Sb^0^ is generated in the presence of PEAI. To further support this observation and clarify the role of PEAI, we performed depth‐profile XPS on SbI_3_‑only treated perovskite sample (Figure S4b). As shown in Figure S4b, no Sb^0^ related peaks were observed in the depth‐profile XPS over time, indicating that PEAI plays a significant role in creating the environment required for the generation of Sb^0^ at the perovskite interface. In addition, to relate the depth‑profile behavior of metallic Sb in the PEAI+Sb device, we carried out XPS measurements on the photoinduced perovskite films (both PVK/PEAI+Sb and PVK/SbI_3_ samples) using AM 1.5G solar simulator. In these experiments, the samples were prepared and subjected to controlled light illumination prior to XPS characterization. Remarkably, as shown in Figure S4c, the photoinduced depth‐profile XPS of PVK/PEAI+Sb shows no significant change as compared to the pristine XPS of PEAI+Sb measured without illumination. In contrast, the PVK/SbI_3_ depth profile (Figure S4d) exhibits only a small reduction of Sb^3+^ to metallic Sb^0^. These observations collectively demonstrate that the presence of PEAI is critical for the in‑situ generation of metallic Sb^0^ by providing a suitable environment at the perovskite interface. It is noteworthy that all XPS characterizations were performed on perovskite thin films fabricated on silicon substrates. Overall, the depth‐profile XPS and photoinduced experiments confirm that metallic Sb^0^ is not formed appreciably in SbI_3_‑only systems but is generated in‐situ only when PEAI is present, underscoring the essential role of the PEAI environment in enabling and stabilizing Sb^0^ at the perovskite surface.

In addition, high‐angle annular dark‐field scanning transmission electron microscopy (HAADF‐STEM) and energy dispersive X‐ray spectroscopy (EDX) analysis were performed to further confirm the presence of the in‐situ generated Sb^0^ on the perovskite surface as shown in Figure S4e–h. STEM images further corroborate the formation of metallic Sb^0^ at the perovskite surface of the PEAI+Sb passivated samples. The lattice fringes with a d‑spacing of ∼0.21 nm can be indexed to the (110) planes of metallic Sb^0^ [41, 42, 43], while the fringes with a d‑spacing of ∼0.315 nm correspond to the (200) planes of the FAPbI_3_ perovskite phase [44, 45, 46]. The simultaneous observation of these two sets of lattice fringes in proximity confirms that Sb^0^ domains are directly embedded at or near the FAPbI_3_ surface. This STEM evidence is fully consistent with our depth‑profile XPS results and provides direct structural confirmation of the in‑situ formation of metallic Sb^0^ in the PEAI+Sb passivated perovskite. In addition, EDX analysis was conducted on the PEAI+Sb treated perovskite films to quantitatively assess the elemental composition and spatial distribution of key elements at the surface of the film. As shown in Figures S4i and S5–S7, EDX spectra and the corresponding elemental mapping images confirm the presence of Sb on the perovskite surface. These results reveal relatively uniform distribution of Sb across the film surface, indicating effective surface coverage rather than localized aggregation, which is significant for achieving homogeneous modification. Furthermore, scanning electron microscopy (SEM) was employed to examine the surface morphologies of the perovskite films. SEM images revealed that the pristine PVK films exhibited a rough surface with uneven textures. Notably, the regions of increased brightness were observed, indicating higher secondary electron emission, which is typically associated with the presence of excess PbI_2_, particularly concentrated at grain boundaries (Figure 1e). In contrast, the films treated with PEAI and PEAI+Sb exhibited compact morphologies, particularly PEAI+Sb treated film displaying a relatively smoother surface and improved grain boundaries and defects.

### 2.2 Interfacial Energy‐Level Alignment and Surface Characterizations

**FIGURE 2:**
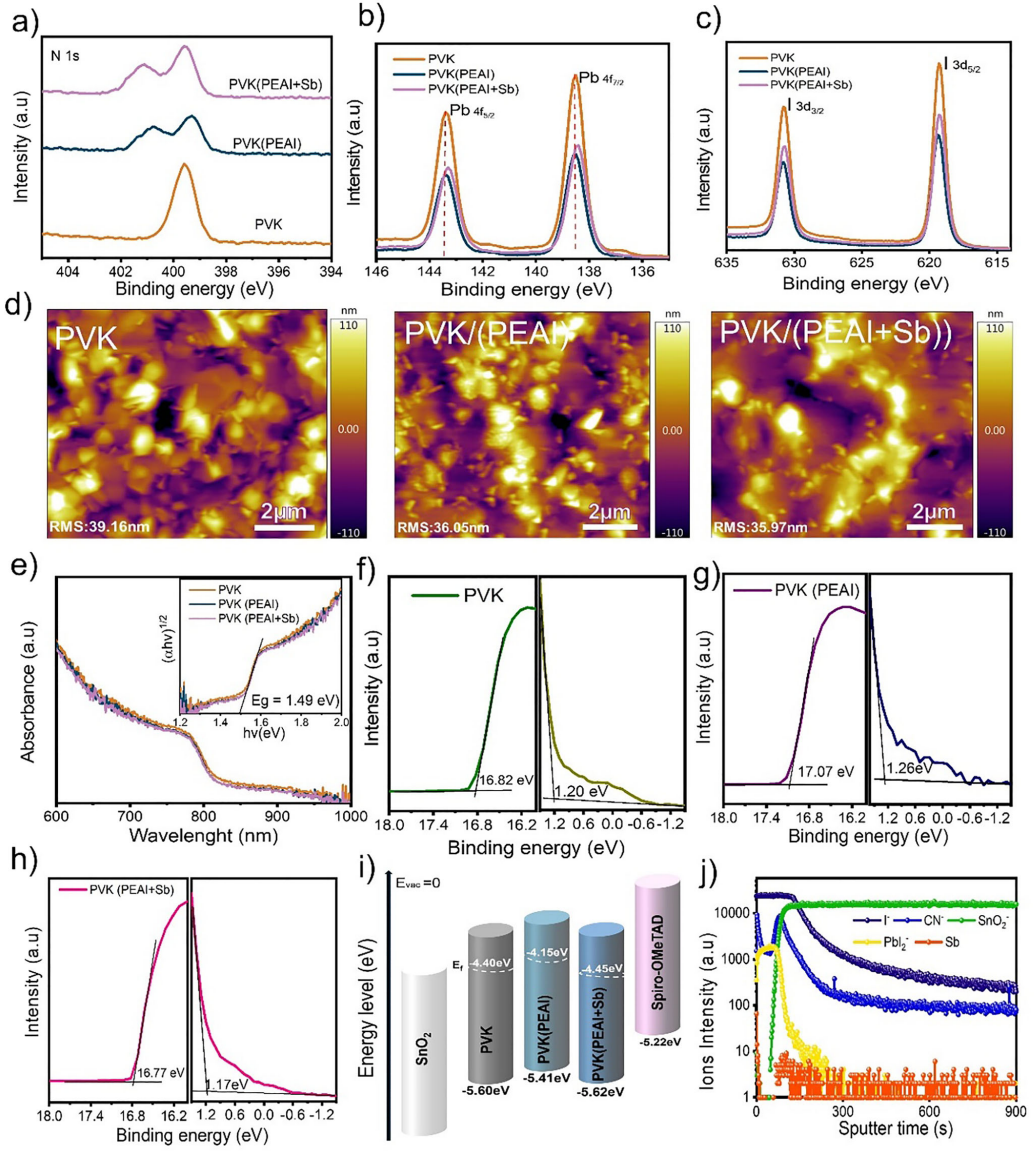
Characterizations of the perovskite films. HR XPS (a) N 1s, (b) Pb 4f, and (c) I 3d spectra of the pristine PVK, PEAI, and PEAI+Sb treated perovskite films and (d) their AFM images. (e) UV–vis absorbance spectra and (f–h) UPS spectra of the PVK, PVK(PEAI), and PVK(PEAI+Sb) films. (i) Energy level diagram and Fermi level of the SnO_2_, perovskite films and Spiro‐OMeTAD. (j) ToF‐SIMS profile of PEAI+Sb treated perovskite films.

Perovskite films treated with PEAI and PEAI+Sb were further subjected to XPS analysis to examine changes in the surface chemical state (Figure S8). As shown in Figure 2a, XPS spectrum of the pristine PVK exhibited a single N 1s peak at 399.6 eV, attributed to the CH = NH_2_^+^/CH‐NH_2_ groups of the formamidinium (FA^+^) cation. After treating with PEAI and PEAI+Sb, additional peaks emerged at 400.7 and 401.1 eV, which correspond to nitrogen species in the phenethylammonium (PEA^+^) cation [47]. The shifts and additional peaks confirm the successful passivation of PEAI on the perovskite surface, altering the surface chemistry by introducing new nitrogen environments. Thus, the presence of these nitrogen peaks in the PEAI+Sb treated films suggests that PEAI remains chemically intact after passivation and Sb incorporation does not adversely affect the chemical state of the organic passivator. Furthermore, as illustrated in Figure 2b, the characteristic Pb 4f XPS peaks of the PEAI+Sb treated film shifted slightly to lower binding energies as compared to the PVK and PEAI treated films. Additionally, the XPS spectra of the I 3d peaks also exhibited similar shifts (Figure 2c). These shifts in the binding energies for both Pb 4f and I 3d suggest strengthened electronic interactions at the interface consistent with Sb‐mediated coordination/passivation of Pb‐I structures, indicating modified local chemical environments and enhanced interfacial bonding in the PEAI+Sb‐treated film in comparison to PVK and PEAI. Both Pb 4f and I 3d spectra shifted to lower binding energies due likely to the space charge effects resulting from changes in the surface work function of the perovskite films [48]. The Pb 4f and I 3d core‐level energy spectra indicate that the pristine PVK film has a Pb:I ratio of 1:2.7, suggesting an iodine deficiency that could lead to defects. After the modifications with PEAI+Sb, the Pb:I ratio increased to 1:3.2, indicating that the perovskite surface had sufficient iodide, likely filling the iodine vacancies (Figure S9) [49].

Additionally, atomic force microscopy (AFM) was employed to assess the surface roughness and the impact of surface passivation on the smoothness of the films. As shown in Figure 2d, AFM images reveal that the PEAI+Sb treated sample showed a noticeably smoother surface as compared to both pristine and PEAI‐treated films. Specifically, the surface roughness was measured to be 39.16 and 36.05 nm for the pristine and PEAI‐treated films, respectively, while the PEAI+Sb treated perovskite film had a surface roughness of 35.96 nm. This reduction in the surface roughness highlights the effectiveness of PEAI+Sb passivation in improving the surface uniformity, which is beneficial for enhancing device stability and performance. The reduction in roughness observed with PEAI+Sb treatment can be attributed to the molecules occupying the smoother areas of the perovskite surface, potentially minimizing defects in lower regions and at the grain boundaries.

Furthermore, ultraviolet photoelectron spectroscopy (UPS) and UV–vis absorption spectroscopy were employed to evaluate the energy level alignment of the films. As shown in Figure 2e, the UV–vis spectra showed no significant shift in the absorption edge for the treated films as compared to the pristine PVK. This indicates that the surface passivation and the introduction of metallic Sb can primarily act as interface modifiers at the perovskite surface, without altering the intrinsic structural or optical properties of the PVK films [50]. The unchanged position of the absorption band maximum in the UV–vis spectra for both PEAI and PEAI+Sb treated films, relative to the pristine PVK film, indicates that the core electronic structure and optical properties of the perovskite materials are conserved after the treatments. This stability is significant because it indicates that the introduction of metallic Sb does not interfere with the intrinsic light absorbing properties of the perovskite films, and thus suggesting that while Sb^0^ enhanced charge transport or modified surface characteristics, it did not compromise the light absorption properties. In addition, the Tauc plots show that the optical bandgaps of the PVK, PEAI, and PEAI+Sb treated films remain to be similar (1.49 eV), indicating that Sb treatment primarily modifies the interfacial properties of the perovskite while effectively reducing non‐radiative recombination and enhancing charge carrier extraction without altering the bandgap. As mentioned above, UPS was used to explore the energy levels of the perovskite films [51]. Figure 2f–h show the UPS spectra of the pristine PVK, PEAI and PEAI+Sb treated films, highlighting both the secondary electron cut‐off and valence band regions. These results show that PEAI post‐treatment changed the Fermi level (E_f_) of the FAPbI_3_ based perovskite film surface from 4.40 to 4.15 eV which is consistent with previously reported values [52, 53]. Interestingly, the PEAI+Sb treatment increased the E_f_ from 4.40 to 4.45 eV; however, this increase is negligible in relation to the vacuum level (E_VAC_ = 0 eV) [51]. As compared to the pristine PVK, the valence band maximum (V_BM_) of the PEAI treated film showed an upward shift from −5.60 to −5.41 eV. On the other hand, the V_BM_ of the PEAI+Sb treated PVK film was measured to be −5.62 eV. The conduction band minimum (C_BM_) values of the PVK, PEAI, and PEAI+Sb treated perovskite films were calculated to be −4.11, −3.92, and −4.13 eV, respectively. Figure 2i shows the energy level alignment of the pristine and treated perovskite films. The shift in the energy band alignment of PEAI+Sb treated perovskite films may facilitate charge carrier extraction and transportation [19, 54]. In addition, we further determined the type of doping (*n*‐type or *p*‐type) introduced by the interface passivation. According to the result, △(E_f_‐CB) for pristine PVK, PEAI and PEAI+Sb treated are 0.29, 0.23, and 0.32 eV, respectively. These results suggest that PEAI and PEAI+Sb treated perovskite films exhibit n‐type and p‐type doping effects, respectively. Therefore, the p‐type modification of the PEAI+Sb treated film could promote the band bending at the perovskite surface [55]. Overall, we confirmed that PEAI and Sb^0^ interface modulation act independently to facilitate the charge extraction and reduce the interfacial charge accumulation. The observed V_BM_ shifts in PEAI and PEAI+Sb treated perovskite films suggest important insights into charge carrier dynamics and energy level alignment [56]. Despite the changes in the V_BM_, the bandgap remains unchanged, indicating that energy levels can be tuned independently offering flexibility for device optimization in optoelectronic and energy applications. To further establish surface passivation with PEAI and interface modulation using Sb, Time‐of‐Flight Secondary Ion Mass Spectrometry (ToF‐SIMS) was conducted to analyze the distribution of ions in the perovskite. As shown in Figure 2j, these measurements reveal a clear distribution of metallic Sb within the perovskite film and an accumulation of Sb near the surface.

### 2.3 Photovoltaic Performance and Charge Carrier Dynamics

**FIGURE 3:**
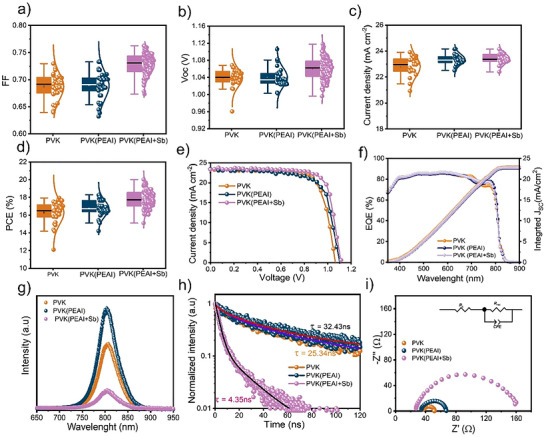
Device and film characterizations. (a) FF, (b) *V*_OC,_ (c) *J*_SC_ and (d) PCE of PVK, PVK(PEAI) and PVK(PEAI+Sb) based PSCs. (e) *J–V* curves and (f) EQE spectra of the champion devices. (g) PL spectra and (h) TRPL decay spectra of PVK, PVK(PEAI) and PVK(PEAI+Sb) films on glass substrates. (i) EIS Nyquist plots of the PVK, PVK(PEAI) and PVK(PEAI+Sb) based devices.

To evaluate the impact of PEAI+Sb treatment on the PV performance, PSC devices with a configuration of FTO/SnO_2_/PVK/Spiro‐OMeTAD/Au were fabricated using different treatments and tested under AM 1.5G illumination. Figure 3a–d display the key PV parameters including open‐circuit voltage (*V*_OC_), fill factor (FF), short‐circuit current density (*J*_SC_), and power conversion efficiency (PCE), while Figure 3e presents the current density‐voltage (*J–V*) curves of the champion devices. The control device without surface passivation exhibited a *J*_SC_ of 23.04 mA/cm^2^, *V*_OC_ of 1.06 V, FF of 0.73, and PCE of 17.93%. The PEAI‐passivated device showed improved performances, with a *J*_SC_ of 23.22 mA/cm^2^, *V*_OC_ of 1.08 V, FF of 0.73 and PCE of 18.27%. Promisingly, the PEAI+Sb‐treated device demonstrated significant improvements, achieving a J_SC_ of 23.39 mA/cm^2^, *V*_OC_ of 1.11 V, and FF of 0.76, yielding a PCE of 20.02%. We attribute these enhanced PV parameters to multiple synergistic factors at the perovskite/hole transport layer (HTL) interface [57, 58]. Specifically, the dynamics of charge collection and extraction during the J‐V scans appear more favorable in the PEAI+Sb treated devices, facilitating efficient charge separation and reducing charge accumulation [59, 60]. On the other hand, to further validate the individual contributions of PEAI and Sb treatment in enhancing charge separation and reducing charge accumulation and recombination at the perovskite/hole transport layer (HTL) interface, we conducted a separate evaluation of devices treated with only Sb. This approach was aimed at isolating the effect of Sb passivation alone to better understand its role in the device. Figure S10 presents comparative statistical data for all PV parameters of the pristine PVK, PEAI‐treated, PEAI+Sb treated, and Sb‐treated devices. The Sb‐treated device achieved a *J*_SC_ of 23.21 mA cm^−2^, *V*_OC_ of 1.08 V, FF of 0.74, and PCE of 18.45%. Both Sb and PEAI‐treated devices show similar performances. This indicates that the synergistic passivation and interfacial modification of perovskite with PEAI and Sb^0^ effectively reduced surface defects and charge recombination at the perovskite/HTL interface. Additionally, as shown in Figure 3f, no significant differences in the external quantum efficiency (EQE) were observed for the three devices, suggesting no changes in the perovskite bandgap and thus resulting in similar *J*_SC_ values, both of which are in excellent agreement with the measured bandgaps of the perovskites and measured *J*_SC_ values. Steady‐state photoluminescence (PL) and time‐resolved photoluminescence (TRPL) spectroscopy were performed to investigate the impact of PEAI and PEAI+Sb treatments on the charge carrier dynamics of the perovskite films. The measurements were conducted on pristine PVK, PEAI‐treated, and PEAI+Sb treated PVK films deposited on glass substrates. The PL intensity of the PEAI‐treated film was slightly higher than that of the control PVK, indicating effective surface modification (Figure 3g). It is well documented that PEAI treatment reduces surface defects, mitigates non‐radiative recombination and improves charge carrier lifetime [24]. On the other hand, the perovskite film (glass/PVK/PEAI+Sb) exhibits significant PL quenching, suggesting that while PEAI effectively passivates surface defects on the perovskite film, the presence of metallic Sb^0^ plays a crucial role in facilitating charge collection and transport to the charge‐selective layer. This dual functionality helps to minimize the surface charge accumulation and significantly reduces the non‐radiative recombination losses, thereby enhancing the overall device performance. Moreover, TRPL measurements reveal that the PEAI+Sb treated perovskite layer exhibits shorter charge carrier lifetimes as compared to both the pristine and PEAI‐only treated PVK films (Figure 3h). This reduction in carrier lifetime is indicative of more efficient charge extraction rather than increased recombination, suggesting that metallic Sb acts as an effective charge mediator at the perovskite surface. Hence, these results reveal that the synergistic impact of combining PEAI passivation with Sb incorporation not only improves the surface quality, but also optimizes the interfacial charge dynamics for high‐performance perovskite optoelectronic devices.

Electrochemical impedance spectroscopy (EIS) was further employed to evaluate the interface properties and help to provide further insights into the charge transport properties of the devices [60, 61]. As shown in Figure 3i, the measured data were fitted using an equivalent circuit model depicted in the inset. The derived Nyquist plots display a single semicircle, which is a characteristic feature of the EIS response for planar n‐i‐p devices [62, 63]. The equivalent circuit consists of a series resistance (*R*_s_), a charge recombination resistance (*R*_rec_), and a constant phase element (CPE) representing the capacitance. These results show that the PEAI+Sb treated devices exhibit a larger semicircle in the EIS plot, corresponding to a higher recombination resistance (*R*_rec_) of 127.20 Ω cm^2^. Meanwhile, the pristine PVK and PEAI treated devices display smaller semicircles with lower *R*_rec_ values of 15.49 and 33.64 Ω cm^2^, respectively. The higher recombination resistance observed in PEAI+Sb treated devices confirms their enhanced ability to suppress charge carrier recombination, leading to improved charge transport and overall device performance as compared to the pristine and PEAI treated counterparts. These findings suggest that the incorporation of Sb^0^ metallic substantially improved the *R*_rec_ of the devices by lowering the carrier recombination, and this further explains phenomenon behind the enhanced *V*_OC_ in PEAI+Sb treated devices.

### 2.4 Effects of PEAI and Sb Metallic Ion Passivation on the Device Stability

**FIGURE 4:**
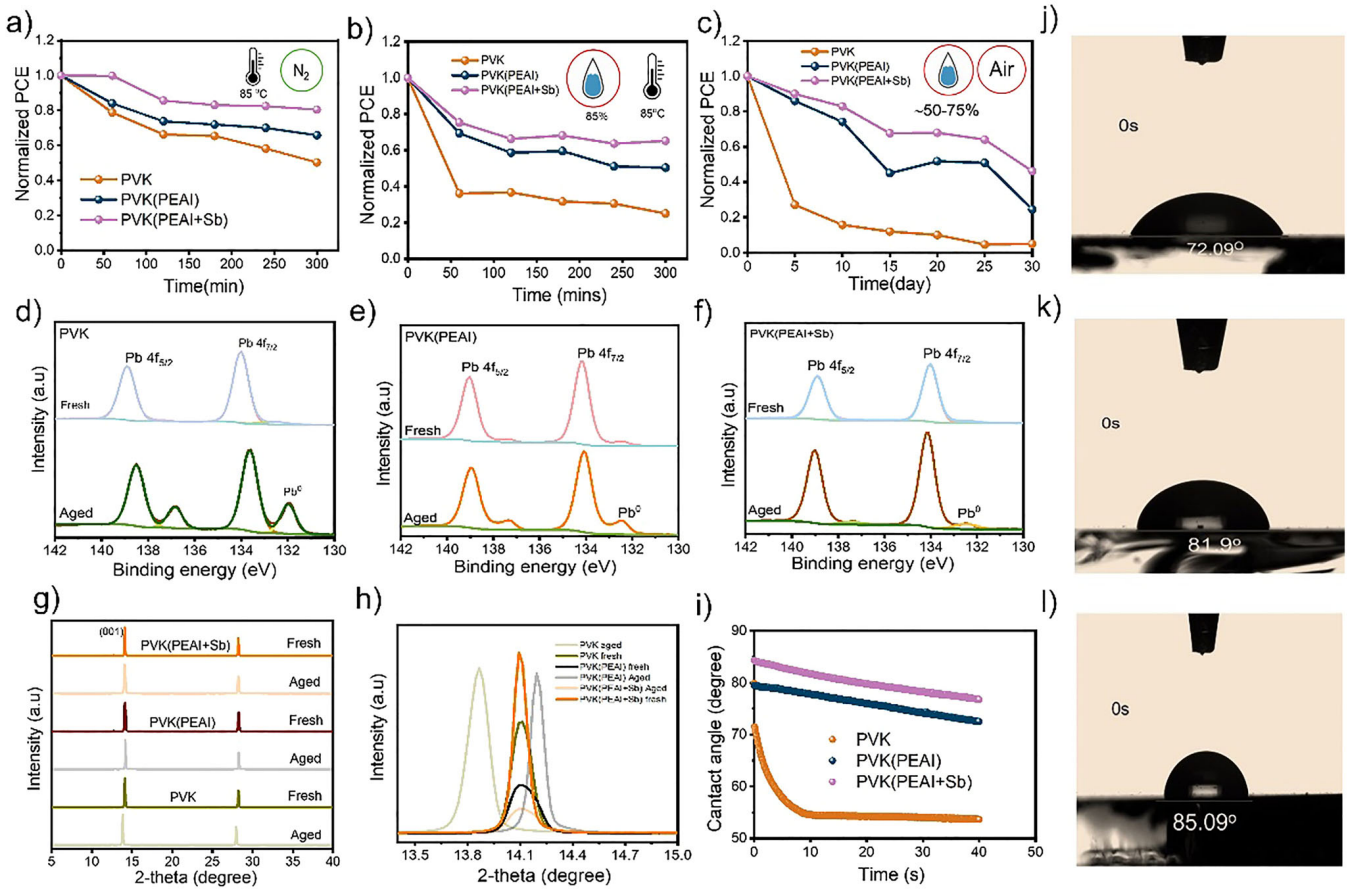
Device stabilities in various conditions. Stability tests of unencapsulated PSCs aged (a) at 85°C under N_2_ atmosphere, (b) at 85°C and 85% RH for 5 h and (c) in ambient condition with ∼50%–75% RH for 30 days. (d–f) XPS Pb 4f spectra of aged and fresh reference, PEAI and PEAI+Sb passivated films at ∼85% RH for 5 h. (g,h) XRD patterns of the perovskite films aged for 30 days in ambient condition with ∼50%–75% RH for 30 days. (i) Average contact angle measurements of perovskite films over 300 s. (j–l) Photograph of water contact angle measurements on the perovskite films.

The lifetime of solar cells is the key to the commercialization of PV technologies. Therefore, we evaluated the damp‐heat stability of unencapsulated PSCs under three different conditions, in accordance with the recommendations from the International Summit on Organic PV Stability [64]. Initially, the reference PSCs without passivation, along with PEAI and PEAI+Sb passivated devices, were stored in a N_2_ atmosphere and annealed at 85°C temperature. Both passivated devices demonstrated comparable stability under these conditions, with PEAI+Sb passivated PSCs retaining 80% of their initial PCEs after 5 h. In contrast, the PEAI‐modified and reference devices maintained only 65% and 50% of their initial PCE, respectively (Figure 4a). This improvement is likely due to the reduced iodine migration from α‐FAPbI_3_ to Spiro‐OMeTAD. On the other hand, the improvement in PEAI+Sb passivated PSCs under the same testing conditions can be attributed to the blocking of the PEAI deprotonation process in the presence of Sb in PEAI. To further justify the statement that the PEAI+Sb passivated device retains over 80% of its initial PCE, we carried out a statistical analysis on a set of 15 working devices. As shown in Figure S11, the results based on both standard evaluation metrics and the maximum efficiency values of the PVK/PEAI+Sb devices show that after 5 h of stability test in ambient conditions at room temperature, the devices retained more than 80% of their initial PCEs. Furthermore, we studied the stability of the devices by annealing devices at 85°C in humid conditions (85% relative humidity (RH)) (Figure 4b). The PEAI+Sb passivated device retained 65% PCE of its initial value, while the PEAI passivated and reference device stored only 50% and 26% of the initial PCEs, respectively, after 5 h. The observed improvement in the stability of the PEAI+Sb passivated device, in comparison to the PEAI passivated device under extreme conditions, indicates that the incorporation of Sb into the PEAI significantly improves the hydrophobicity of the perovskite surface (Figure 4b). In addition, the increased hydrophobicity from Sb modification protects the perovskite from moisture by reducing water affinity and helps to mitigate the harmful effects of humidity, preventing degradation and enhancing the device durability and efficiency. Furthermore, a long‐term stability test was conducted on unencapsulated devices under ambient conditions (∽50%–75% RH) for 30 days. As shown in Figure 4c, the PEAI and PEAI+Sb passivated devices retained approximately 24.5% and 46.5% of their initial PCEs, respectively. However, the pristine device without any passivation exhibited a significantly lower retention (only 5.2%), indicating that the PEAI+Sb passivation strategy provides superior stability. Therefore, enhanced stability can be attributed to the improved resistance against moisture‐induced degradation, which promotes prolonged device performance under ambient conditions.

To further confirm the stability of PEAI+Sb modified device, a long‐term stability test was conducted using an ISOS‐compliant stability tester (LitosLite, Fluxim). In accordance with the ISOS‐L‐1 protocol, maximum power point (MPP) tracking was performed in air under continuous simulated one‐sun illumination. As shown in Figure S12, the encapsulated PEAI+Sb passivated devices retained ∼57.67% of their initial PCE after 500 h of continuous test. On the other hand, the PEAI‐only passivated devices displayed faster degradation, retaining ∼49.15% of the initial PCE after 500 h under identical conditions. These long‐term MPP tracking results are consistent with our short‐term 85°C stress test and confirm that the PEAI+Sb passivation improves the operational stability of the devices relative to PEAI alone and pristine perovskite. Furthermore, we analyzed the chemical composition of the pristine, PEAI, and PEAI+Sb passivated films using XPS after aging. Compared to the freshly prepared perovskite films, the aged samples demonstrated distinct new features in the Pb 4f high‐resolution (HR) XPS spectra. Specifically, prominent peaks corresponding to metallic lead (Pb^0^) were observed in all aged perovskite films (Figure 4d–f). This indicates partial reduction of Pb^2+^ to Pb^0^ during aging, which is typically observed in metal halide perovskite films [65]. However, the intensity of these metallic Pb^0^ peaks varied between the differently passivated devices, providing insight into the effectiveness of passivation treatments in mitigating degradation pathways. The measured ratios of metallic lead (Pb^0^) to Pb^2^^+^ were found to be 26% for the PEAI‐passivated films, 13% for the PEAI+Sb passivated films, and 46% for the reference (non‐passivated) perovskite films. This further indicates the extent to which lead ions in the perovskite material have been reduced from their original Pb^2^^+^ state to metallic Pb^0^ due to the aging. To further corroborate the findings obtained from XPS analysis, X‐ray diffraction (XRD) characterization was performed on both fresh and aged perovskite films for the reference, PEAI and PEAI+Sb passivated samples (Figure 4g,h). The characteristic XRD peak corresponding to the 001 plane of the aged PEAI+Sb passivated perovskite film remained unchanged. But the 001 plane of the aged PEAI passivated perovskite film shifted slightly to a higher angle with increased intensity. However, the aged reference α‐FAPbI_3_ perovskite film showed a significant shift to a lower angle, accompanied by broader and reduced intensity after being stored in ambient conditions for more than 30 days. The results suggest that incorporating Sb into PEAI significantly decreases its high reactivity with the perovskite surface, and consequently preventing its rapid conversion into PEA_2_PbI_4_, which could lead to substantial degradation of α‐FAPbI_3_ perovskite film [67], and thus facilitating a structurally more stable PEAI+Sb passivated perovskite film.

To further corroborate the observed stability enhancements, the water contact angles of the reference, PEAI and PEAI+Sb passivated perovskite films were measured (Figure 4i,j–l). The initial contact angles for the reference, PEAI, and PEAI+Sb passivated films were 72.09°, 81.09°, and 85.09°, respectively. These values decreased to 53.63°, 72.37°, and 76.94°, respectively. These results show that the incorporation of Sb into the PEAI layer effectively enhances the hydrophobicity of the perovskite surface, thereby reducing the moisture penetration. The higher initial and sustained contact angles observed for the PEAI+Sb passivated films indicate a significant improvement in surface water repellences as compared to both the reference and PEAI passivated films. The result suggests that the presence of Sb within the PEAI passivation layer reduces the reactivity at the perovskite interface and inhibits the deprotonation of PEAI molecules [66]. Therefore, this dual effect helps to stabilize the perovskite structure and minimizes the degradation pathways, which often originate from moisture‐induced interface reactions.

**FIGURE 5:**
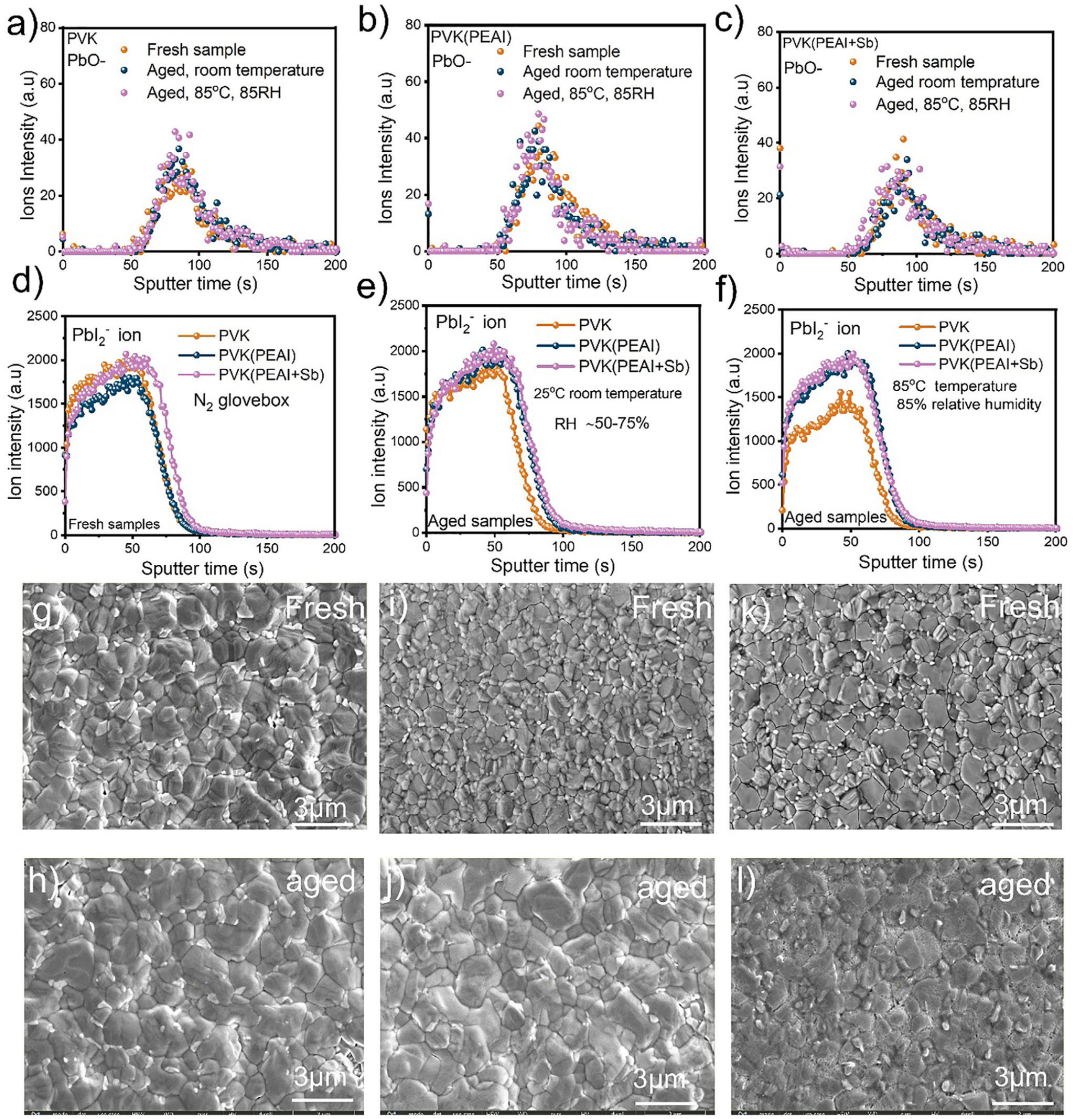
ToF‐SIMS and SEM characterizations of fresh and aged perovskite films. PbO^−^ ion spectra of (a) fresh reference PVK, PVK aged at room temperature (25°C) and RH of ∽50%–75%, and PVK aged at 85°C temperature and 85% RH, (b) fresh PVK(PEAI) in, PVK(PEAI) aged at room temperature (25°C) and RH of ∽50%–75%, and PVK(PEAI) aged at 85°C temperature and 85% RH (c) fresh PVK(PEAI+Sb), PVK(PEAI+Sb) aged at room temperature (25°C) and RH of ∽50%–75%, and PVK(PEAI+Sb) aged at 85°C temperature and 85% RH. PbI_2_^−^ ion spectra of (d) fresh reference PVK, PVK/PEAI and PVK(PEAI+Sb), (e) reference PVK, PVK/PEAI and PVK(PEAI+Sb) aged at room temperature (25°C) and RH of ∽50%–75%, and (f) reference PVK, PVK/PEAI and PVK(PEAI+Sb) aged at 85°C temperature and 85% RH. SEM images of fresh and aged samples: (g,h) pristine PVK, (i,j) PVK(PEAI), and (k,l) PVK(PEAI+Sb) films. The aging experiment was carried out in room temperature and ∽50%–75% RH.

To further elucidate the role of Sb incorporation into the PEAI, we conducted ToF‐SIMS measurements on fresh and aged samples. The measurements were performed on the film architectures comprising FTO/SnO_2_/PVK, FTO/SnO_2_/PVK(PEAI), and FTO/SnO_2_/PVK(PEAI+Sb). Figure 5a–f shows the depth profiles of PbO^−^ and PbI_2_^−^ ion concentrations for the FTO/SnO_2_/PVK, FTO/SnO_2_/PVK(PEAI), and FTO/SnO_2_/PVK(PEAI+Sb) samples, as revealed by ToF‐SIMS, where each concentration profile has been normalized by the total ion counts within the SnO_2_ region where the compositions should be identical (see Figure S13a–c). As shown in Figure 5a–c, the depth profiles of PbO^−^ for the fresh and aged FTO/SnO_2_/PVK, FTO/SnO_2_/PVK(PEAI), and FTO/SnO_2_/PVK(PEAI+Sb) samples reveal clear differences in PbO^−^ concentrations between the different samples. Notably, the FTO/SnO_2_/PVK(PEAI+Sb) sample maintains a consistently lower PbO^−^ concentration, both after aging under ambient room conditions and when aged at 85°C and 85% RH. In contrast, the FTO/SnO_2_/PVK and FTO/SnO_2_/PVK(PEAI) samples tend to exhibit higher PbO^−^ accumulation under the harsh humidity and temperature stress. These results indicate that the incorporation of PEAI+Sb effectively suppresses perovskite oxidation, thereby mitigating degradation and substantially enhancing device stability. In addition, Figure 5d–f present ToF‐SIMS depth profiles of PbI_2_^−^ for fresh FTO/SnO_2_/PVK, FTO/SnO_2_/PVK(PEAI), and FTO/SnO_2_/PVK(PEAI+Sb) samples. In all fresh samples, the Pb‐related signal remains relatively constant with increasing sputtering time, indicating uniform composition through the film thickness prior to stress. After aging under ambient conditions (room temperature, ∽50%–75% RH) and after accelerated aging (85°C and 85% RH for 5 h), noticeable changes were observed. In the reference FTO/SnO_2_/PVK, the PbI_2_^−^ signal drops significantly after aging. In contrast, the FTO/SnO_2_/PVK(PEAI+Sb) displayed nearly unchanged PbI_2_^−^ signal under both aging conditions. Specifically, as highlighted in Figure 5f for the 85°C/85% RH test, the pristine perovskite (FTO/SnO_2_/PVK) exhibits a pronounced decline in PbI_2_^−^ intensity toward the surface, evidencing rapid degradation. Meanwhile, both passivated devices, especially FTO/SnO_2_/PVK(PEAI+Sb), show negligible change in PbI_2_^−^ concentration profile shape when compared to the unaged samples. These results indicate that PEAI+Sb treatment delivers the most robust suppression of perovskite decomposition across the film thickness.

In addition, the ToF‐SIMS profiles of iodide (I^−^) ions (Figure S13d–f) exhibit trends consistent with those of PbO^−^ and PbI_2_^−^. For fresh films, the concentration profiles of I^−^ in pristine PVK, PVK(PEAI), and PVK(PEAI+Sb) are similarly shaped; however, the PVK(PEAI+Sb) peak intensity is noticeably higher than that of pristine PVK and PVK(PEAI) (Figure S13d). This indicates that the fresh PVK(PEAI+Sb) films prepared in the N_2_ glovebox possess a higher iodine content without signs of degradation, corroborating the stronger I 3d signal observed in the XPS. Upon aging under ambient conditions (room temperature, ∼50%–75% RH), the I^−^ signal in PVK(PEAI+Sb) is measured with a peak intensity similar to pristine PVK and PVK(PEAI), pointing to enhanced resistance to degradation (Figure S13e). Furthermore, following accelerated aging at 85°C/85% RH as shown in Figure S13f, the I^−^ peak intensity in PVK(PEAI+Sb) shows no significant increase, whereas pristine PVK exhibits a pronounced rise in I^−^ signal compared with the passivated PVK(PEAI) and PVK(PEAI+Sb) films. This behavior is characteristic of non‐passivated perovskites, where abundant grain boundaries, surface defects, and undercoordinated Pb^2+^ sites serve as fast ion‐migration pathways. Under harsh aging, I^−^ ions segregate and accumulate at interfaces, amplifying iodine‐related signals and facilitating the formation of iodine‐rich phases. In contrast, the significantly lower I^−^ intensity in aged PVK(PEAI+Sb) agrees with the suggestion that the PEAI+Sb treatment forms an interfacial passivation layer that suppresses iodine migration and passivates undercoordinated Pb^2+^ and halide vacancies. Iodine remains more uniformly distributed and less prone to aggregation. The absence of such passivation in control PVK leaves more active migration channels, leading to greater iodine redistribution and iodine‐rich phase formation, and thus a higher iodine signal upon aging.

As shown in Figure S14a, the XPS evidence of Pb‐O formation shows that fresh control film shows only a weak broad O 1s signal attributable to adsorbed species ≈531.5‐534 eV (Figure S14a). However, after aging, a new obvious O 1s component appears at ≈529.5–530.5 eV (Figure S14b), which is characteristic peak of lattice oxygen in Pb‐O environments (PbO_x_). These oxide‑related features grow with aging time and are accompanied by an initial increase in PbI_2_ peak, supporting a degradation sequence in which perovskite first decomposes to PbI_2_ and is then oxidized to PbO_x_. In contrast, PEAI+Sb treated films show strongly suppressed Pb‐O components in XPS and reduced PbO_x_‑related fragments in ToF‑SIMS as compared to fresh perovskite and PEAI passivated films, confirming that the PEAI+Sb interfacial passivation effectively retards the formation of lead oxides on the perovskite surface.

The comprehensive ToF‐SIMS analysis presented here provides crucial insights into the degradation mechanisms and elemental stability of perovskite thin films under varied environmental stresses. Our results clearly demonstrate that the incorporation of Sb into the PEAI layer substantially enhances the resistance of perovskite films from degradations under both moderate (room temperature, ∽50%–75% RH) and accelerated (85°C, 85% RH) aging conditions. These findings highlight the effectiveness of incorporating Sb in reducing a key degradation pathway in perovskite optoelectronic devices. Mechanistically, the synergistic PEAI+Sb passivation suppresses the oxidation of perovskite into lead oxides through combined chemical, electronic, and physical effects. PEAI+Sb treatment passivates undercoordinated Pb^2^^+^ and halide vacancies at the surface, reducing the density of highly reactive sites that initiate perovskite decomposition to PbI_2_ and subsequent oxidation to PbO_x_. On the other hand, the HR XPS N 1s results show that Sb stabilizes the protonated PEA^+^ species under aging (Figure S14), while depth‐profile XPS reveals the in‑situ formation of interfacial Sb^0^, which, together modifies band bending and screens surface charge. This mitigates the formation of reactive oxygen species responsible for oxidizing PbI_2_. In addition, the thin phenethylammonium layer acts as a partial barrier to O_2_/H_2_O ingress. Consistent with this mechanism, the aged control films exhibit pronounced Pb‐O peak in XPS (Figure 4d) and strong PbO_x_‑related ToF‑SIMS signals (Figure 5a), whereas these signatures are strongly suppressed in PEAI+Sb treated films.

In addition, to assess the morphological and surface changes of the PVK, PVK(PEAI), and PVK(PEAI+Sb) films after aging, SEM characterizations were performed on both fresh and aged samples. As shown in Figure 5g–l, substantial morphological changes were observed in the unmodified PVK and PVK(PEAI) after aging. It should be noted that the samples for SEM analysis were aged at room temperature and ∽50%–75% RH for three weeks. In contrast, the PVK films modified with PEAI+Sb displayed notably smoother and uniform surface after undergoing the same aging process. The preservation of surface morphology in the PEAI+Sb modified samples strongly suggests enhanced stability and resistance to atmospheric degradation under these conditions. These SEM results highlight the effectiveness of Sb incorporation in combination with PEAI in mitigating surface deterioration and maintaining the structural integrity of perovskite films during extended exposure to ambient humidity.

Furthermore, to directly probe the proposed suppression of PEAI deprotonation by PEAI+Sb passivation, we performed N 1s XPS measurements on PEAI and PEAI+Sb passivated perovskite films before and after aging under the same ambient conditions (room temperature, ∽50%–75% RH). As shown in Figure S15, all perovskite films (PVK, PVK/PEAI and PVK/PEAI+Sb) exhibited a N 1s component at ∼399–400 eV, which is typically observed in organic‐inorganic halide perovskites as well as many organic passivation materials including PEAI. Nevertheless, for the freshly prepared PEAI passivated films, the N 1s spectrum displays a pronounced high binding energy peak at ∼401–402 eV, characteristic of protonated ammonium (‐NH_3_^+^) in PEA^+^ in addition to its peak at ∼399–400 eV. After aging in air, the PEAI passivated samples showed significantly reduced peak at 401–402 eV binding energy. This change in the N 1s spectra can be interpreted as a partial deprotonation of PEA^+^. In contrast, the PEAI+Sb films retain a predominantly –NH_3_^+^ character even after aging, with only minor changes in the relative intensities of both ∼401–402 eV and 399–400 eV components, indicating that Sb co‐passivation effectively suppresses PEA^+^ deprotonation. Concurrent XPS Pb 4f analysis reveals that the aged non‐passivated and PEAI passivated perovskite films develop a pronounced low binding energy shoulder associated with Pb^0^ species, whereas this feature is strongly suppressed in the PEAI+Sb samples (Figure 4d–f). These observations demonstrate that Sb incorporation stabilizes both the protonation state of the PEAI and the perovskite layer, thereby inhibiting the coupled deprotonation reduction pathway that otherwise contributes to surface degradation and performance loss. These results indicate that Sb incorporation effectively stabilizes the interfacial chemistry of PEAI‐passivated perovskites. Specifically, PEAI+Sb preserves the protonated ‐NH_3_^+^ state of PEA^+^ and maintains Pb in the Pb^2^^+^ state after aging, in contrast to PEAI‐only films, which show PEA^+^ deprotonation and Pb reduction. Consequently, the PEAI+Sb strategy suppresses the coupled deprotonation reduction pathway that accelerates surface degradation and device performance loss. As compared to previously reported dual‑molecular passivation strategies, which generally combine two organic cations or ligands that both predominantly heal halide vacancies and induce similar surface potential changes [19, 67, 68, 69], the PEAI+Sb strategy presented here is chemically and mechanistically distinct. Our findings reveal that PEAI mainly coordinates with under‑coordinated Pb and A‑site related terminations to form a stabilized organic capping layer, meanwhile, metallic Sb^0^ acts as a local charge reservoir, partially shielding the charge and altering the interfacial band alignment.

### 2.5 Theoretical Calculations

**FIGURE 6:**
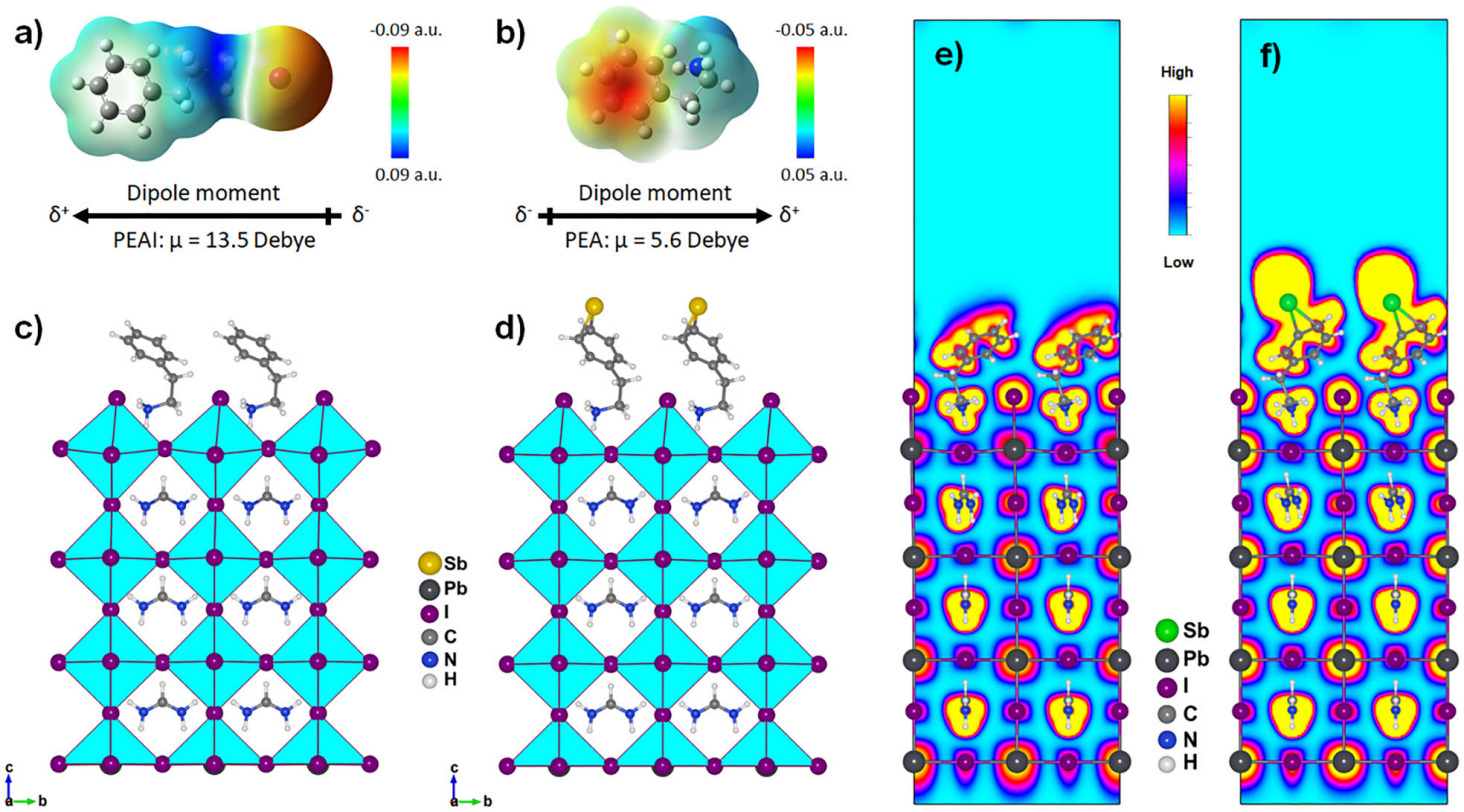
Theoretical results of PVK(PEAI) and PVK(PEAI+Sb) FAPbI_3_ systems. Calculated dipole moments and EPS maps of (a) PEAI and (b) PEA. Optimized crystal structures of (c) PVK(PEAI) and (d) PVK(PEAI+Sb) FAPbI_3_ (001) slabs. ELF plots for (e) PVK(PEAI) and (f) PVK(PEAI+Sb) FAPbI_3_ slabs.

To explore the atomistic origin of intrinsic electronic properties of the phenylethylammonium iodide (PEAI) and phenylethylammonium (PEA) molecules, we first conducted quantum chemistry calculations based on molecular density functional theory (DFT) using Gaussian16 software (Figure 6; Figure S16). Figure 6a,b show the calculated electronic polarity of PEAI and PEA, visualized by GaussView6 software. The result shows that PEAI exhibits a much stronger dipole moment of 13.5 Debye due to the presence of the iodide counter‐ion (Figure 6a), while neutral PEA has a dipole moment of 5.6 Debye (Figure 6b). We envisaged that this pronounced polarity could allow PEAI to interact effectively with the PbI_2_‐terminated FAPbI_3_ surface and may lead to considerable surface passivation. Quantum solid‐state first‐principles calculations based on periodic DFT were then performed using the Vienna Ab initio Simulation Package (VASP) code to understand the interaction of PEAI and Sb additives with the crystalline FAPbI_3_ perovskite surface at the atomic level. Details of the simulations are provided in the supporting information.

At first, we investigated the interaction between the PEAI additive and the pristine PbI_2_‐terminated α‐FAPbI_3_ (001) surface (PVK). Figure 6c shows the optimized crystal structure of PEAI‐treated FAPbI_3_ perovskite PVK(PEAI) slab. Structural optimization calculation reveals that PEAI does not merely adsorb onto the PVK surface; instead, it interacts with the PVK slab through a curious dual‐pronged mechanism. First, the iodide of PEAI, after optimization, is directly bound to the undercoordinated Pb atom of the pristine PVK surface, forming a robust Pb‐I coordination bond. The emerging chemical Pb‐I bond complements the local octahedral chemical environment of Pb, passivating the undercoordinated Pb surface sites. Second, the PEA of PEAI also serves an important role. The result indicates that the PEA molecule occupies the void near the surface, passivating the FA‐like sites at the surface (Figure 6c). Hence, this dual mechanism, where iodine passivates undercoordinated Pb sites while PEA passivates FA‐like sites, creates a stabilizing capping layer at the surface that improves the stability of the perovskite lattice.

We further investigated how the Sb‐additive interacts with the PEAI‐treated FAPbI_3_, aiming to understand the synergistic effects of incorporating Sb and PEAI additives onto the FAPbI_3_. As shown in Figure 6d, the structural optimization calculation reveals that the Sb atomic additive preferentially binds to the carbon atoms of the phenyl ring within PEA rather than bonding directly with the FAPbI_3_ surface. The adsorption energy is relatively high at –1.7 eV. Such a high negative value implies a strong binding interaction and a stable PVK (PEAI + Sb) system.

Moreover, the electron localization function (ELF) plot reveals that the PEAI+Sb capping additive induces significant charge redistribution at the surface (Figure 6e,f) that can be beneficial for further charge transport. The result also quantitatively suggests that the incorporation of Sb acts as a structural stabilizing anchor that reinforces the PEA and locks its orientation in place, thereby strengthening the capping layer. This enhanced interfacial rigidity can improve the stability of the perovskite surface. Our theoretical findings indicate that the combination of PEAI and Sb treatments presents promising sequential and synergistic strategies for passivating the perovskite surface while enhancing charge modulation. Notably, the incorporation of additional Sb offers an extra degree of freedom, facilitating charge transport. This metalloid‐ligand (Sb‐phenyl) synergistic design principle can potentially be applied to other systems using similar metalloids (e.g., Sn, Bi, In). In addition, Figure S17 shows a direct relationship between the structural geometry and electronic density of states (DOS). When Sb binds to the PEAI benzene ring (Figure S17a), new electronic states are generated in the DOS plot (Figure S17c). Notably, the V_BM,_ originating from electronic states hybridizations of Sb and PEAI, shifts toward the Fermi‐level compared to the PVK case as shown in Figure S17d. This result indicates that Sb+PEAI leads to “*p*‐type doping” behavior, which is consistent with our experimental UPS measurements. Such behavior of PVK (PEAI+Sb) can promote interfacial charge transfer and could optimize the band alignment.

## 3 Conclusions

In summary, we have demonstrated an effective strategy for enhancing PSC performance through the in‐situ deposition of metallic antimony (Sb^0^) combined with PEAI, serving as synergistic interfacial charge modulators and passivators on the perovskite surface. The incorporation of Sb into PEAI effectively suppresses deprotonation at the interface while providing efficient defect passivation. Additionally, the generation of metallic Sb^0^ species, characterized by its lower oxidation state, plays a pivotal role in modulating the surface energetics of the perovskite and facilitating the charge transport properties. This optimizes interfacial electron extraction, reduces charge accumulation, and suppresses non‐radiative recombination losses at the interface. Importantly, the device with PEAI+Sb treatment demonstrated significantly enhanced thermal stability, retaining over 80% of its initial PCE after 5 h of continuous heating at 85°C under inert atmosphere conditions. In addition, according to our theoretical calculations, PEAI's larger dipole (13.5 D vs. 5.6 D for PEA) drives strong Pb‐I coordination and effective passivation of undercoordinated Pb on PbI_2_‐terminated FAPbI_3_, preserving the bandgap. On the other hand, Sb preferentially binds to the PEA phenyl ring, yielding a stable interfacial configuration. Overall, PEAI and Sb synergistically stabilize the surface and enhance interfacial charge transport, highlighting a practical route to high performance PSCs. This strategy offers a promising pathway for developing high‐performance and durable solar cells through synergistic interfacial modifications.

## Conflicts of Interest

The authors declare no conflicts of interest.

## Supporting information




**Supporting File**: advs75208‐sup‐0001‐SuppMat.docx.

## Data Availability

The data that support the findings of this study are available from the corresponding author upon reasonable request.
