# Supplementary material for: Interfacial Engineering by Metallic Ions and Organic Ammonium Ligand Passivation for Perovskite Solar Cells
Source: Adv Sci (Weinh). 2026 Apr 9:e20257. Online ahead of print. doi: 10.1002/advs.202520257 (PMC13334653; doi:10.1002/advs.202520257)
Supplement: Supplementary file 1 — Supporting File: advs75208‐sup‐0001‐SuppMat.docx. [file ADVS-9999-e20257-s001.docx]

**Supporting Information** for

**Interfacial Engineering by Metallic Ions and Organic Ammonium Ligand Passivation for Perovskite Solar Cells**

*Abraham Adenle, Purevlkham Myagmarsereejid, Selengesuren Suragtkhuu, Tuul Tsagaantsooj, Solongo Purevdorj, Eric Campbell,* *Sina Jamali, Oisín E. FitzGerald, Taylor J. Z. Stock, Thomas J. Macdonald,* *Joseph F.S. Fernando, Hui Jin, Paul E. Shaw, Michitoshi Hayashi, Batjargal Sainbileg, Chihaya Adachi, Yu Lin Zhong and Munkhbayar Batmunkh**

A. Adenle, P. Myagmarsereejid, S. Suragtkhuu, S. Purevdorj, E. Campbell, S. Jamali, Y. L. Zhong, M. Batmunkh

School of Environment and Science, Griffith University, Nathan, Queensland 4111, Australia

Email: [m.batmunkh@griffith.edu.au](mailto:m.batmunkh@griffith.edu.au)

T. Tsagaantsooj, C. Adachi

Center for Organic Photonics and Electronics Research (OPERA), Kyushu University, Fukuoka, 819-0395 Japan

O. E. FitzGerald, T. J. Z. Stock, T. J. Macdonald

Department of Electronic and Electrical Engineering, University College London, Roberts Building, Torrington Pl, WC1E 7JE, London, United Kingdom

T. J. Z. Stock

London Centre for Nanotechnology, University College London, 19 Gordon St, WC1H 0AH, London, United Kingdom

J. F. S. Fernando

Centre for Microscopy and Microanalysis, The University of Queensland, St Lucia, Queensland 4072, Australia.

School of Chemistry and Physics and Centre for Materials Science, Queensland University of Technology (QUT), 2 George St, Brisbane, Queensland 4000, Australia

H. Jin, P. E. Shaw

Centre for Organic Photonics & Electronics, School of Chemistry and Molecular Biosciences, The University of Queensland, Brisbane, QLD, 4072, Australia

M. Hayashi, B. Sainbileg

Centre for Condensed Matter Sciences, Centre of Atomic Initiative for New Materials, National Taiwan University, Taipei 106, Taiwan

**Experimental section**

**Materials**

All chemicals and reagents were used without further purification. Unless otherwise stated, all chemicals were purchased from Sigma-Aldrich. Formamidinium iodide (FAI), methylammonium chloride (MACl) and 2,2′,7,7′-tetrakis[N,N-di(4-methoxyphenyl)amino]-9,9′-spirobifluorene (Spiro-OMeTAD) were obtained from Xi’an Polymer Light Technology Co. Lead (II) iodide (PbI_2_) was purchased from Thermo Fisher Scientific, Phenethylammonium iodide (PEAI) was purchased from Greatcell Solar Materials.

**Preparation of formamidinium lead triiodide (FAPbI_3_)**

Black α-formamidinium lead triiodide (α-FAPbI_3_) was synthesized by mixing 3.36 g of FAI with 9 g of PbI_2_ in a 1:1 molar ratio and dissolved in 11 mL of 2-ME by stirring. The mixed solution was heated to 120°C in an oil bath and stirred for 45 min. Subsequently, the precipitated FAPbI_3_ was filtered using filter paper (Whatman) without cooling to room temperature (RT). The filtered FAPbI_3_ was baked on a hot plate at 150°C (in air) for 30 min and then left overnight in a vacuum chamber at room temperature (RT).

**Chemical Bath Deposition (CBD) of SnO_2_ layer**

The fluorine-doped tin oxide (FTO) substrate was cleaned by washing with wipes in detergent solution, and sonicating in DI water, acetone, and isopropanol (IPA) for 15 min each. The CBD solution was prepared by mixing 625 mg urea, 625 μL HCl, 12.5 μL TGA, and 137.5 mg SnCl_2_·2H_2_O in 50 mL DI water. One edge of the FTO substrates were taped with Kapton tapes to prevent the deposition of SnO_2_. The FTO substrates and CBD solution were added to a reaction vessel and subjected to a 90°C reaction for three hours. We used a Hellendahl staining dish with a vessel capacity of approximately 170 mm. 50 mL of the CBD solution was applied to eight FTO substrates that had been positioned vertically inside the jar. Upon completion of the reaction, the SnO_2_ deposited FTO substrates were removed from the reaction vessel and cleaned via sonication with DI water and IPA for 5 min each.

**Device fabrication**

The obtained FTO/SnO_2_ films were annealed in an ambient environment (RH 30-40%) at 180°C for 60 min, followed by depositing 10 mM KCl in DI water at 3,000 rpm for 30 s sec (2,000 rpm/sec ramp) and annealing at 100 °C for 5 min. Then, the KCl deposited FTO/SnO_2_ substrates were treated with ozone and then transferred into a nitrogen glovebox. The perovskite precursor solution was prepared by dissolving 1.4 M FAPbI_3_ powder with MDACl_2_ (3.8 mol%) and MACl (35 mol%) in a mixed solvent of DMF: DMSO (8:1 volume ratio). The perovskite solution was spin-coated onto the substrate at 1,000 rpm for 10 s and 5,000 rpm for 30 s (both 2,000 rpm/sec ramp). At 18 seconds into the 5,000 rpm setting, 1,000 μL diethyl ether solution was dropped onto the substrate. Afterwards, the FTO/SnO_2_/perovskite films were annealed at 160^o^C for 15 min in ambient condition with (RH ~40%.) For the perovskite passivation, a 15 mM PEAI solution in IPA was spin coated at 5,000 rpm onto the obtained FTO/SnO_2_/FAPbI_3_. For surface passivation involving Sb, 15 mM PEAI was mixed with 8 mM antimony (III) iodide (SbI_3_) in IPA, followed by spin coating onto the surface of FTO/SnO_2_/FAPbI_3_ at 5,000 rpm for 30 s.

The hole transporting layer (HTL) was deposited by spin coating the HTL solution, consisting of 90 mg Spiro-OMeTAD, 39 μL tBP and 23 μL Li-TFSI solution (520 mg mL^-1^ in acetonitrile) in 1 mL chlorobenzene, at 3000 rpm for 20 sec with a ramp of 2000 rpm/sec. The HTL solution preparation and deposition were performed inside a nitrogen glovebox. Then, Au electrode (70 nm) was deposited by thermal evaporation.

**Characterization details**

Scanning electron microscopy (SEM) images were acquired using a field-emission-scanning electron microscope (JOEL 7100 F). X-ray diffraction (XRD) patterns were recorded using a on a Rigaku Smart lab diffractometer equipped with a 9 kW Cu rotating anode operating at 45 kV and 200 mA with a scanning range from 5° to 60° (2θ). Atomic force microscopy (AFM) was performed in air using Asylum Research Cypher S with Asylum Research software, operating in standard tapping mode configuration using AIR cantilever holder***.*** X-ray photoelectron spectroscopy (XPS) and ultraviolet photoelectron spectroscopy (UPS) data were acquired using a Kratos Axis Supra+ using an Al Kα (λ = 1486.6 eV) and He I (λ = 21.22 eV) source, respectively. Base pressure in the analysis chamber was 1.0 × 10^-9^ torr and during sample analysis 1.0 × 10^-8^ to Current density-voltage (J-V) curves were recorded using Ossila J-V test system under 1 sun illumination (AM 1.5 G, 100 mW cm^-2^) with a scan rate of 125 mV s^-1^. The active area of the tested solar cells was defined by a metal mask with an aperture area of 0.10 cm^2^. The illumination intensity was 100 mW cm^-2^ from the solar simulator (Sun 2000, Abet Technologies), which was calibrated using a National Renewable Energy Laboratory (NREL) certified standard 2 cm × 2 cm silicon photodiode.

Electrochemical impedance spectroscopy (EIS) measurements were carried out on a potentiostatic excitation applied frequencies between 10^-1^ and 10^6^ Hz under illumination using Gamry electrochemical workstation (Interface 1010E). The spectra were fitted using a ZView software. All the measurements were performed at room temperature without encapsulation. Contact angle measurements carried out apparent contact angle using an optical tensiometer (Theta Flex, Biolin Scientific, Finland) and drop size was 5 µL recorded contact duration for 300 s.

The absorption spectra were recorded using a Agilent 8453 spectrophotometer. The steady-state Photoluminescence (PL) emissions were recorded using spectrometer (Hamamatsu Photonics PMA12) to record the PL spectra with the third harmonic (355 nm) of an Nd-YAG laser (EKSPLA, PL-2250) as the excitation source. A streak camera (Hamamatsu Photonics, C10910) was used to measure the transient PL and the third harmonic (355 nm) of an Nd-YAG laser (EKSPLA, PL-2250) was used as the excitation light. TRPL measurements were recorded under vacuum with the time range of 120 ns. External quantum efficiency (EQE) was conducted using a quantum efficiency system (IQE 200B, Newport).

**Stability test using ISOS-L-1 protocol**

The devices were encapsulated using a two-part A-B epoxy. Encapsulation was carried out in a nitrogen atmosphere at 25 °C by sealing the device with a top glass cover followed by edge sealing. The encapsulated devices were left in the glovebox overnight to ensure complete curing. Device stability was evaluated using an ISOS-compliant stability tester (LitosLite, Fluxim). The devices were loaded in the test holder with a glass cover. In accordance with the ISOS-L-1 protocol, maximum power point (MPP) tracking was performed in air under continuous simulated one-sun illumination provided by a light-emitting diode (LED) lamp without a UV filter. It is noteworthy that we observed pronounced initial drops in the PCEs of both PEAI and PEAI+Sb passivated devices. After this rapid decline, the efficiency briefly stabilized near its maximum value. This behaviour is typically observed and known as “burn-in” loss, likely associated with interfacial or chemical instabilities under bias and illumination. Therefore, we have discarded the stability data of initial 20 h in both devices.

**Theoretical calculations**

Quantum first-principles calculations were performed using the Vienna Ab-Initio Simulation Package (VASP, v.5.4.4) with projector augmented wave (PAW) pseudopotentials.^1,2^ The exchange-correlation functionals of spin-polarized Perdew–Burke–Ernzerhof (PBE) functional including Grimme's D3 correction (PBE-D3) and Heyd-Scuseria-Ernzerhof (HSE06) hybrid functional were applied for calculations of structural and electronic properties. The spin-orbit coupling (SOC) was considered. The 520 eV energy cut-off for the plane-wave and Monkhorst–Pack 8 × 8 × 1 k-points were utilized in all calculations. PbI₂-terminated FAPbI₃ (001) perovskite surfaces were constructed with three molecular layers by cleaving the optimized cubic bulk structure. In the slab structure, a vacuum region thicker than 20 Å was added in the direction perpendicular to the surface of FAPbI_3_ perovskite to avoid any artificial influence between adjacent slabs. All structures were optimized until the convergence criteria for the electronic self-consistent energy, and the atomic forces were below 0.1 µeV and 1 meV/Å, respectively.

The adsorption energy ($E_{\mathrm{ads}}$) is estimated using the following equation:

$$E_{\mathrm{ads}}=E_{\mathrm{system}}- E_{\mathrm{surface}}- E_{\mathrm{ad}}$$

where $E_{\mathrm{system}}$, $E_{\mathrm{surface}}$, and $E_{\mathrm{ad}}$ are the total energies of the PVK(PEAI+Sb) system, the PVK(PEAI) surface, and the Sb species, respectively. The negative value of $E_{\mathrm{ads}}$ indicates a more energetically favourable system.


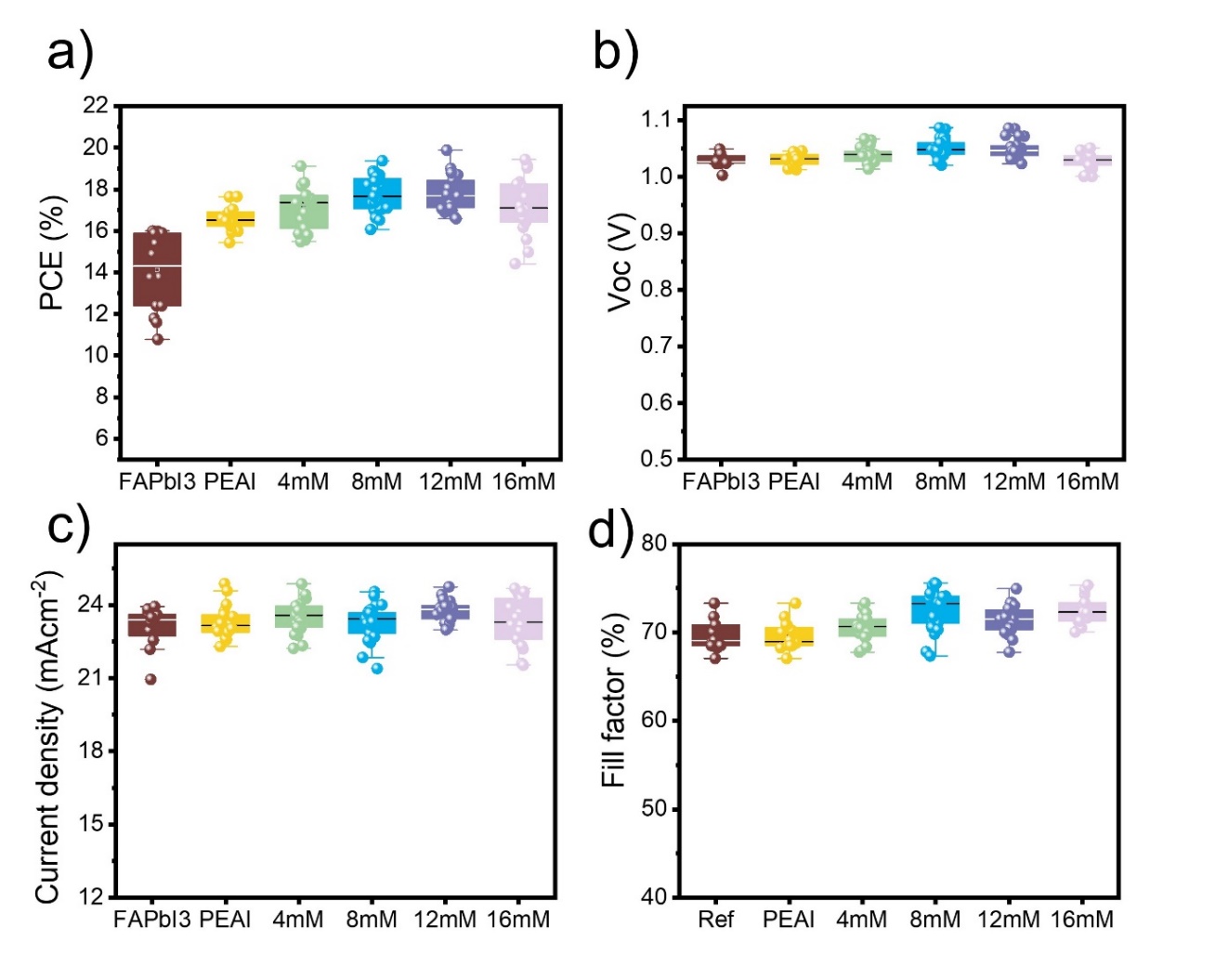


**Figure S1.** Statistical distribution of photovoltaic parameters of perovskite solar cells (PSCs) with different concentrations of SbI_3_: a) power conversion efficiency (PCE), b) open-circuit voltage (V_oc_) c) short-circuit current (J_sc_) and d) Fill factor (FF).

**Figure S2.** High-resolution (HR) Sb *3d* XPS spectra of the pristine, PEAI, PEAI+Sb and Sb-only treated perovskite films.


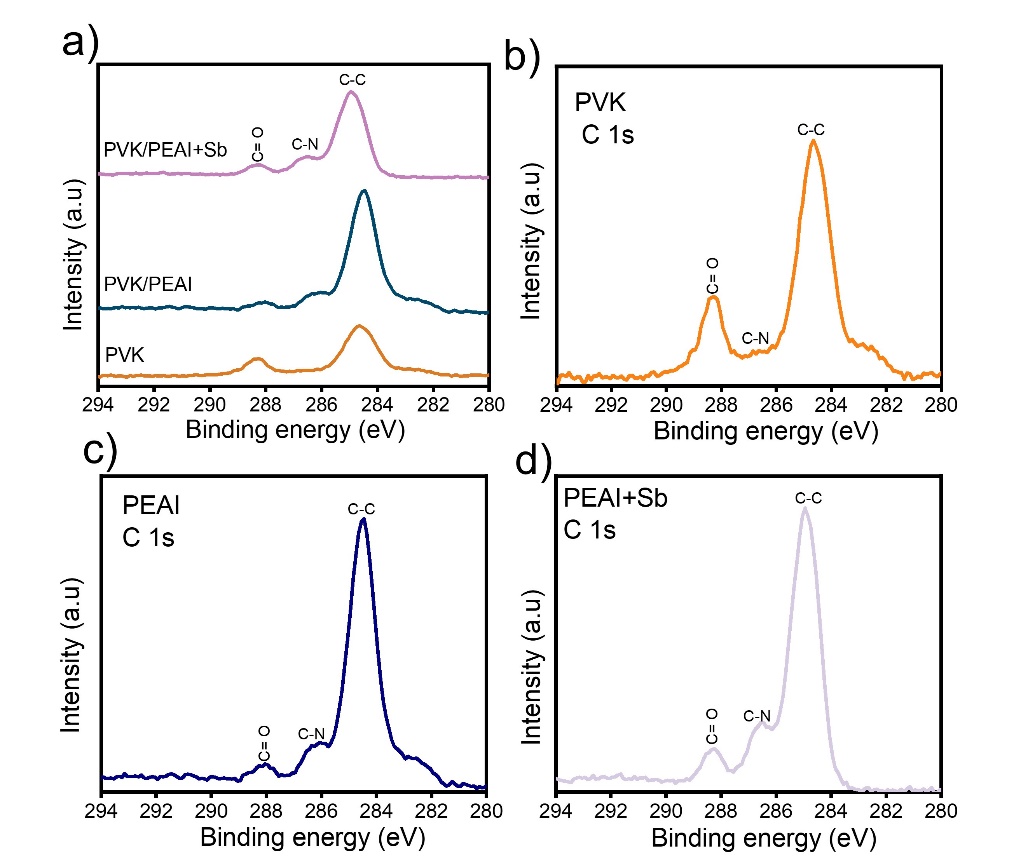


**Figure S3**. a-d) HR XPS C 1s core level spectra of the pristine PVK, PEAI, and PEAI+Sb treated perovskite films.

*
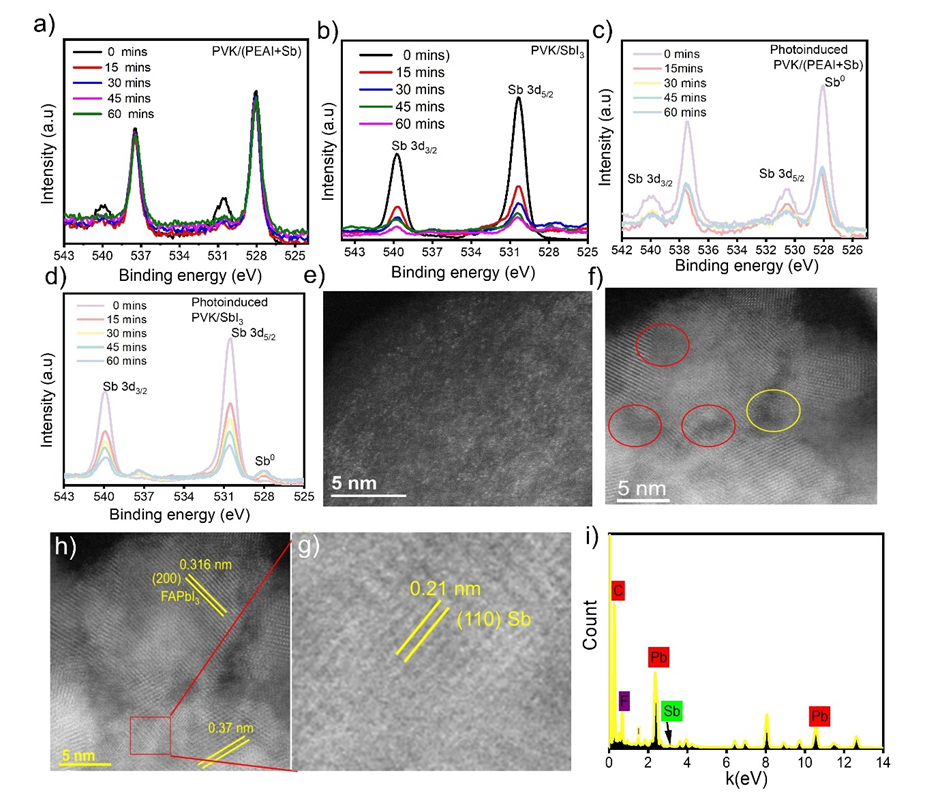
*

**Figure S4.** Time‑resolved depth-profile XPS measurements of a) Sb in PEAI+Sb passivated perovskite film, b) Sb in PVK/SbI_3_ perovskite film, c) Sb in photoinduced (for 2 hrs) PEAI+Sb passivated perovskite film, d) Sb in photoinduced (for 2 hrs) SbI_3_-only treated perovskite film. e-h) HRTEM images of Sb on perovskite. i) EDX spectrum of PVK/PEAI+Sb.


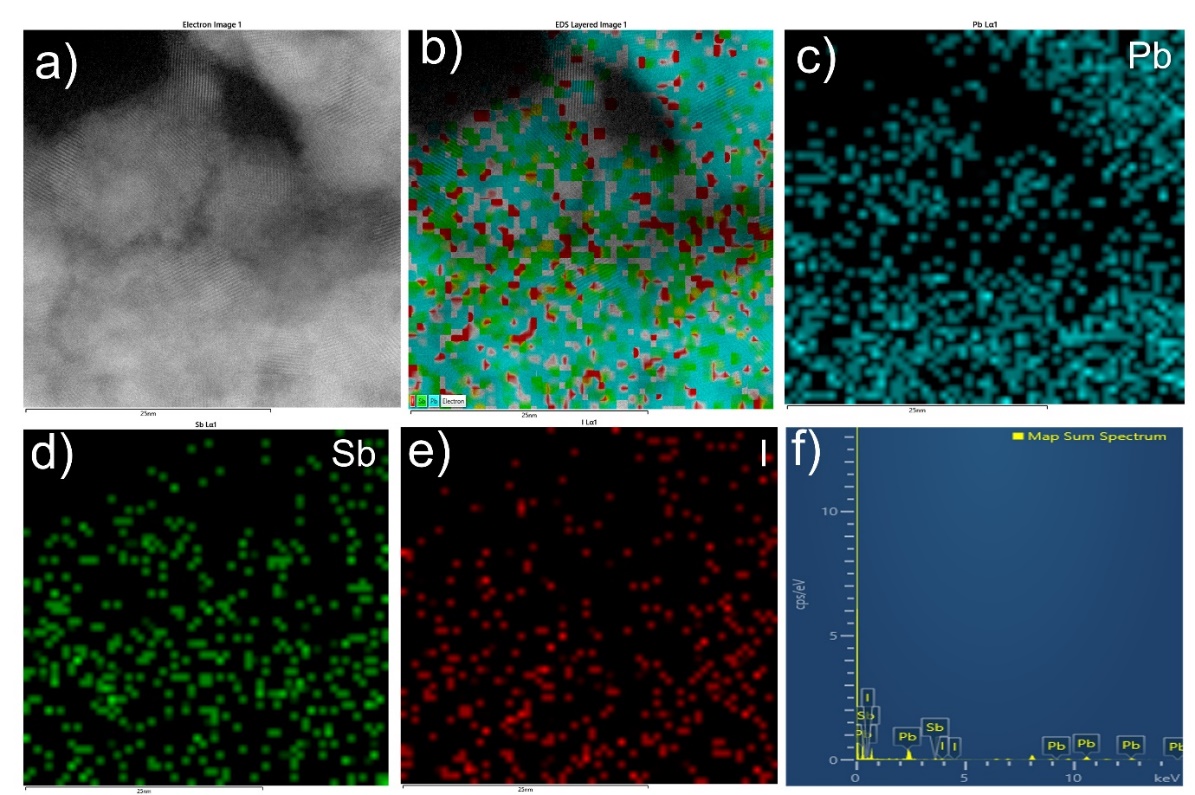


**Figure S5.** a-e) EDX elemental mapping images of PEAI+Sb passivated perovskite films and (f) the corresponding EDX spectrum.


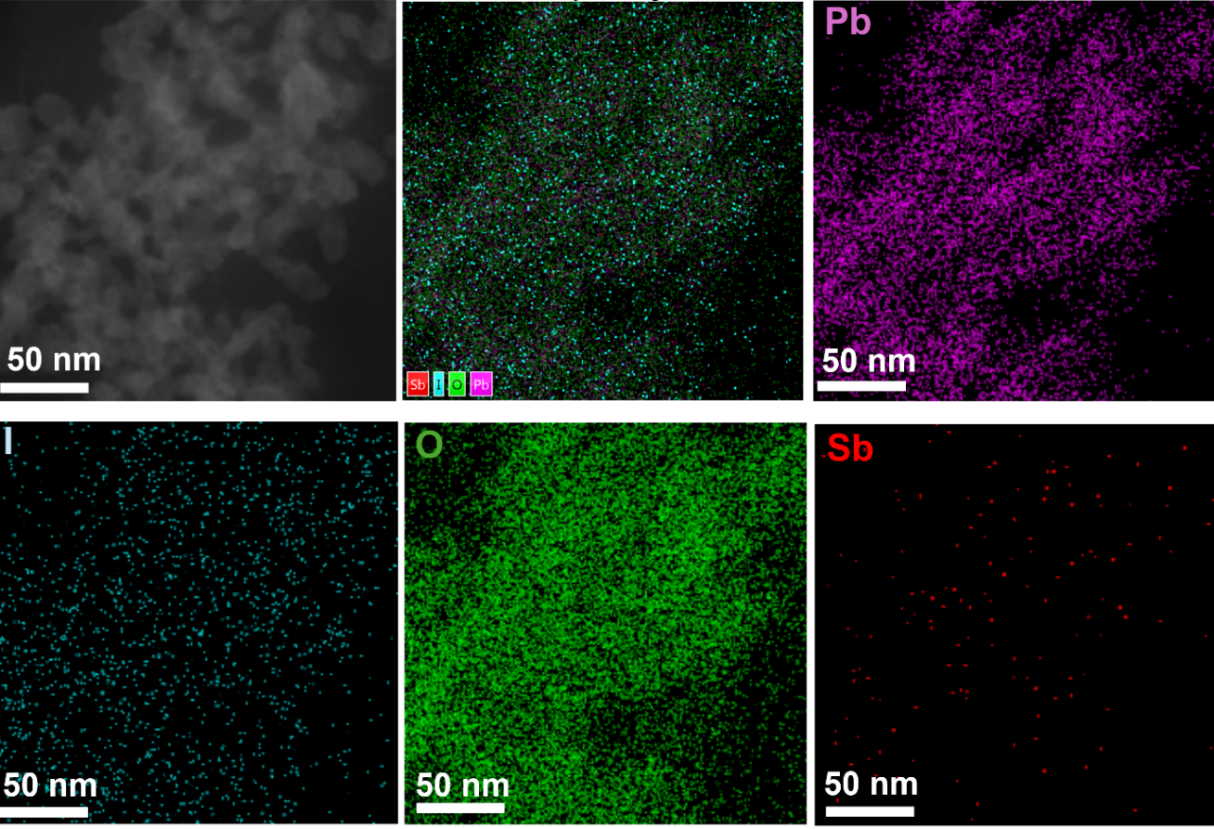


**Figure S6.** High- resolution transmission electron microscopy (HRTEM) and the corresponding elemental mapping images of PEAI+Sb treated perovsksite film.


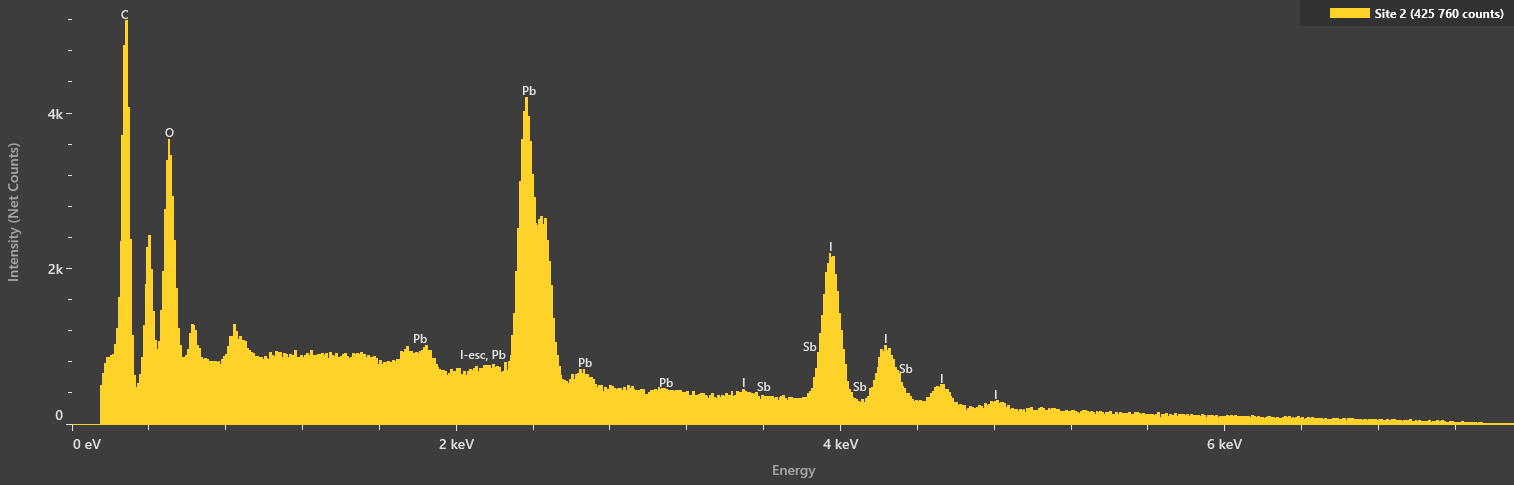


**Figure S7**. EDX spectrum of the PEAI+Sb treated perovskite films.


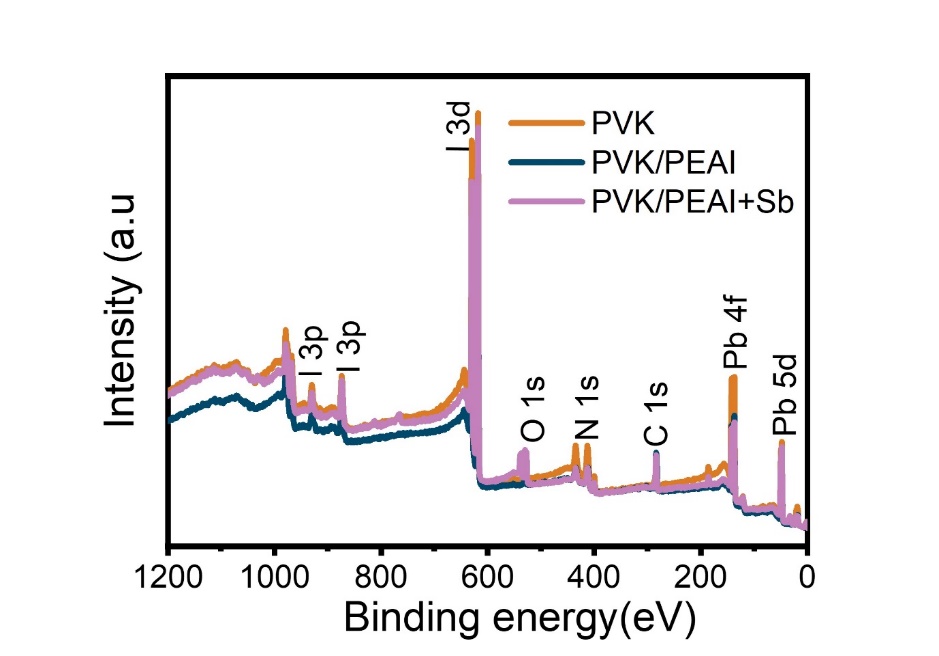


**Figure S8**. Survey XPS scan of the pristine, PEAI and PEAI+Sb treated perovskite samples.


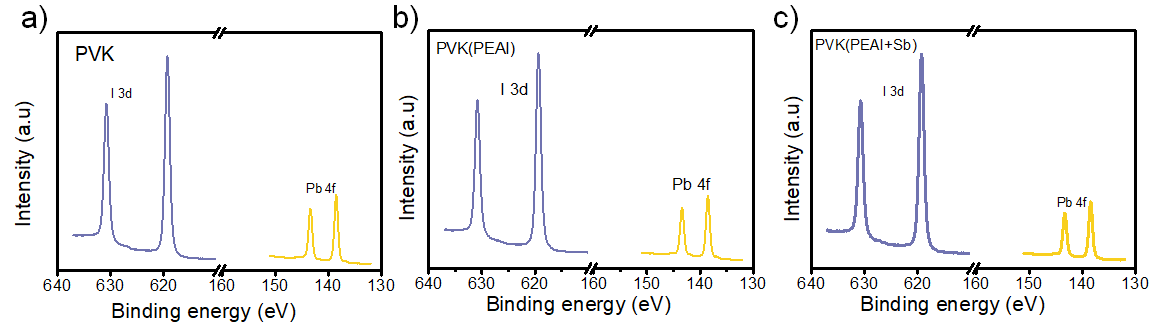


**Figure S9.** XPS Pb 4f and I 3d core energy level spectra of a) PVK, b) PVK(PEAI) and c) PVK(PEAI+Sb). It can be calculated that the ratio of Pb/I are 1:2.7, 1:2.9 and 1:3.2 for PVK, PVK(PEAI) and PVK(PEAI+Sb), respectively.


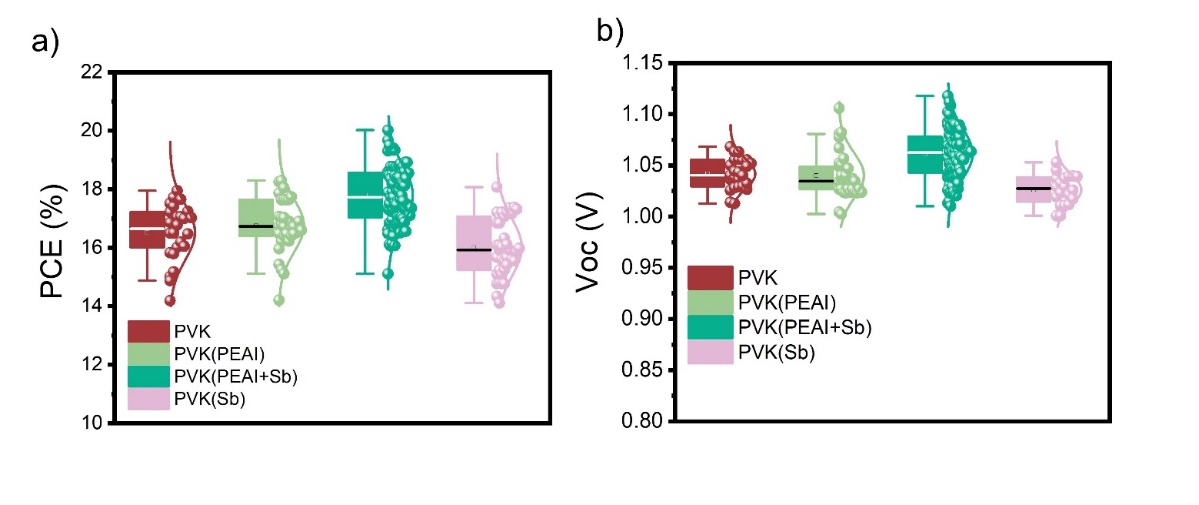


**Figure S10.** Statistical distribution of photovoltaic parameters based on pristine, PEAI PEAI+Sb and Sb-only treated perovskite based solar cells: a) PCE and b) V_oc_.

**Figure S11**. Statistical stability analysis of PEAI+Sb passivated 15 PSCs tested over 5 hours in ambient conditions at room temperature (∽50-75% RH).


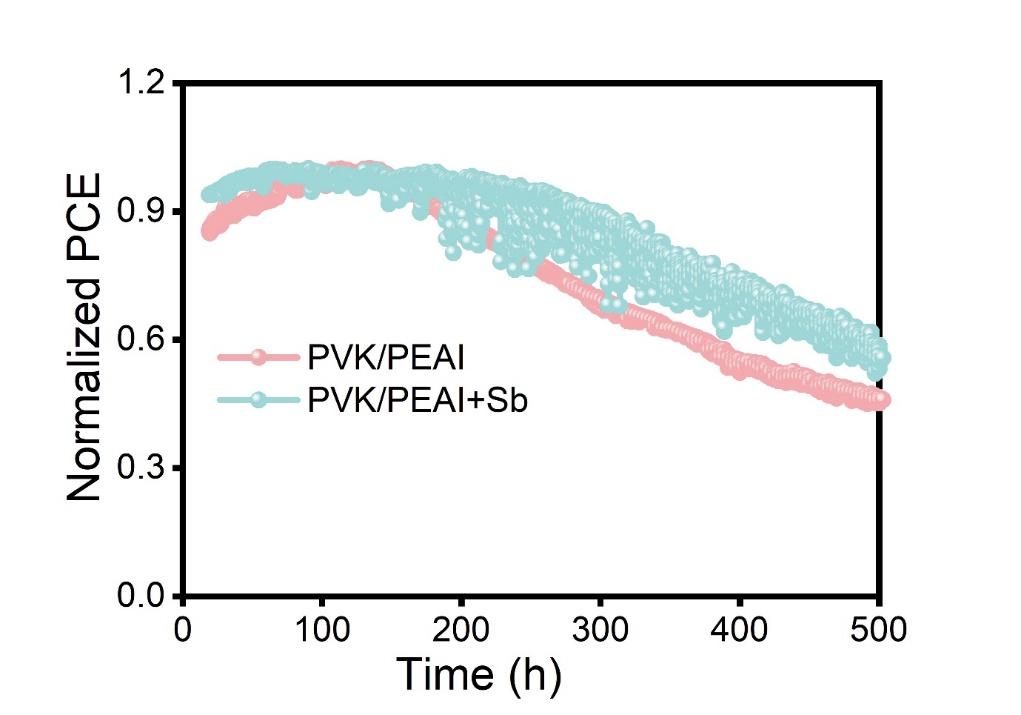


**Figure S12.** Long-term stability test of PEAI and PEAI+Sb passivated PSCs. The devices were encapsulated using a two-part A-B epoxy. The stability test was performed in accordance with the ISOS-L-1 protocol, maximum power point (MPP) tracking was performed in air under continuous simulated one-sun illumination provided by a light-emitting diode (LED) lamp without a UV filter.


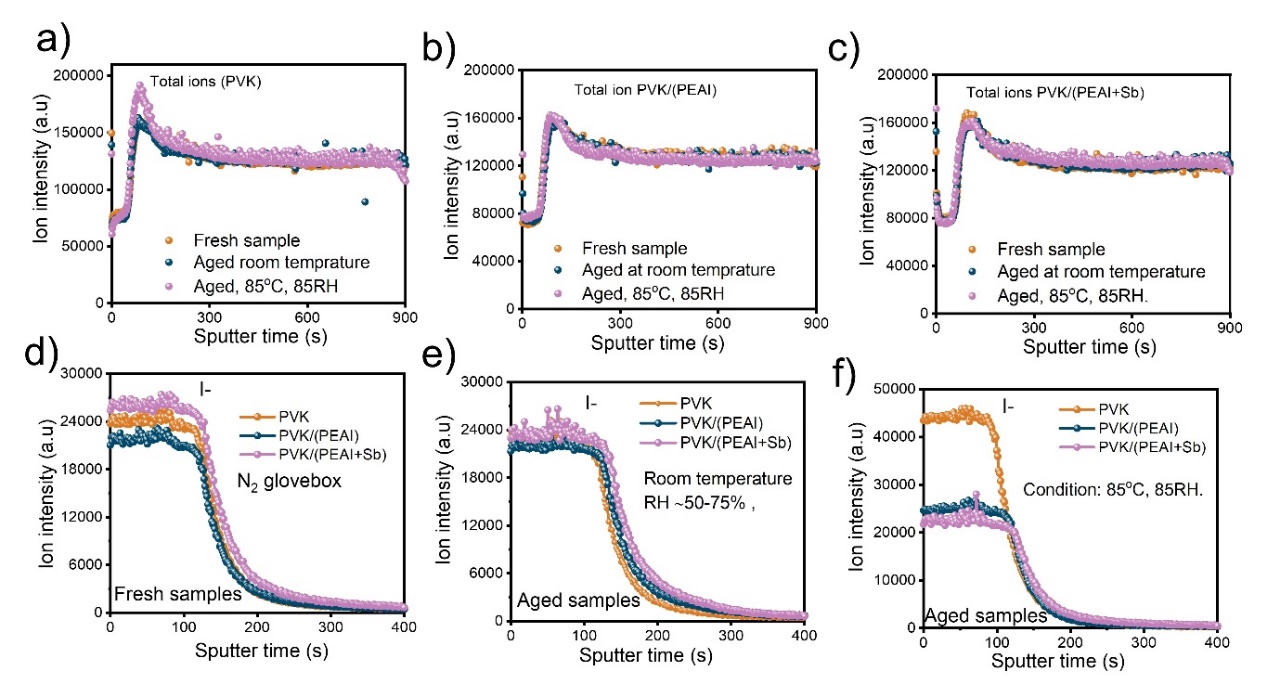


**Figure S13**. ToF-SIMS spectra of the perovskite films. Total ion spectra of a) fresh reference PVK, PVK/PEAI and PVK(PEAI+Sb), b) reference PVK, PVK/PEAI and PVK/(PEAI+Sb) aged at room temperature (25^o^C) and RH of approximately ∽50-75%, and c) reference PVK, PVK/PEAI and PVK/(PEAI+Sb) aged at 85^o^C temperature and 85% RH. Iodide (I^-^) spectra of d) fresh reference PVK, PVK/PEAI and PVK/(PEAI+Sbs), e) reference PVK, PVK/PEAI and PVK/(PEAI+Sb) aged at room temperature (25^o^C) and RH ∽50-75%, and f) reference PVK, PVK/PEAI and PVK/(PEAI+Sb) aged at 85^o^C temperature and 85% RH.


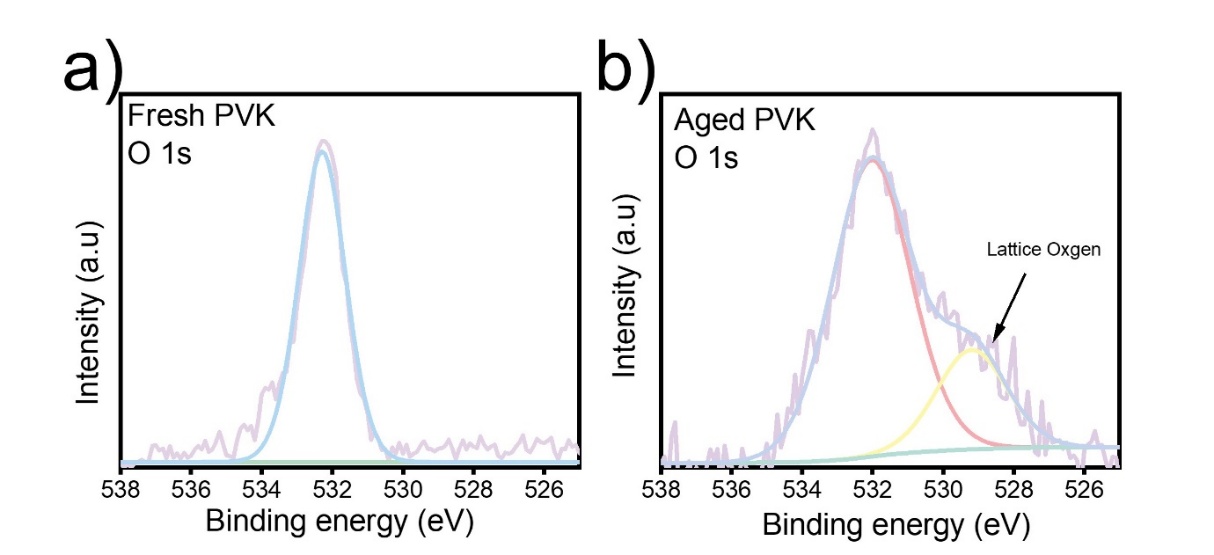


**Figure S14.**  HR XPS O 1s spectra of a) the pristine control PVK, and b) the aged pristine control PVK (~50-75% RH for 15 days).


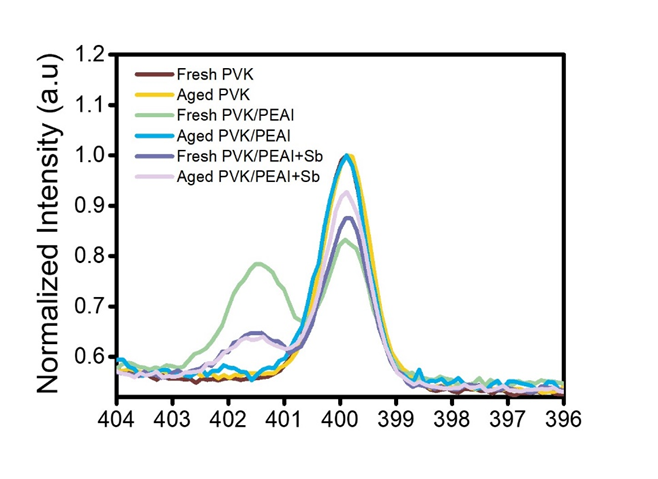


**Figure S15.** XPS N 1s spectra of PVK, PVK/PEAI and PVK/PEAI+Sb passivated perovskite films before and after aging in ambient condition for 30 days (∽50-75% RH).


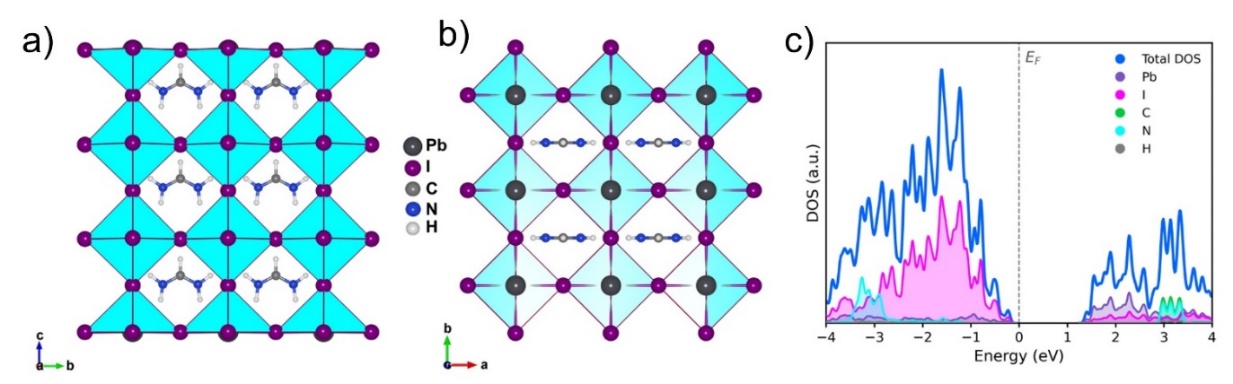


**Figure S16.** Optimized structure of pure FAPbI_3_ (001) PVK slab.


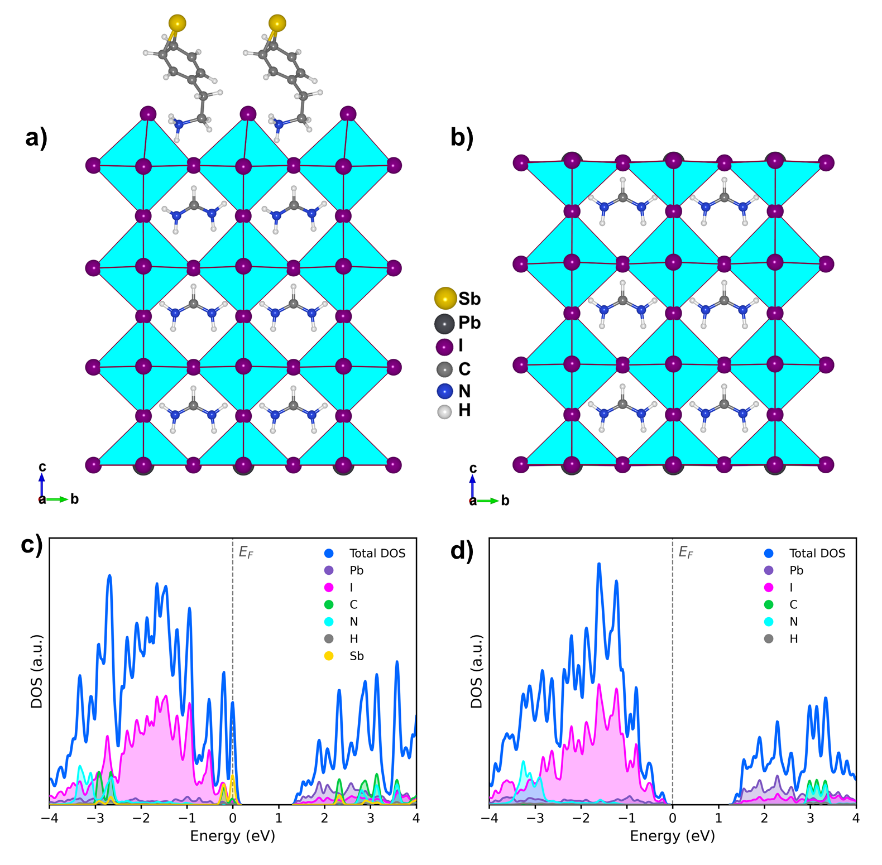


**Figure S17**. Optimized crystal structures of a) PVK(PEAI+Sb) and b) pure PVK FAPbI_3_ (001) slabs, and their electronic DOS plots for c) PVK(PEAI+Sb) and d) pure PVK slabs.

**References**

1. Kresse, G. & Furthmüller, J. Efficient iterative schemes for ab initio total-energy calculations using a plane-wave basis set. Phys Rev B Condens Matter 54, 11169-11186 (1996). https://doi.org/10.1103/physrevb.54.11169

2. Perdew, J. P., Burke, K. & Ernzerhof, M. Generalized Gradient Approximation Made Simple. Phys Rev Lett 77, 3865-3868 (1996). https://doi.org/10.1103/PhysRevLett.77.38655
